# The future of education equity policy in a COVID-19 world: a qualitative systematic review of lessons from education policymaking

**DOI:** 10.12688/openreseurope.13834.1

**Published:** 2021-07-08

**Authors:** Paul Cairney, Sean Kippin

**Affiliations:** 1 History, Heritage, and Politics, University of Stirling, Stirling, Stirling, FK94LA, UK

**Keywords:** Policymaking, Education, Equity, Race, Complexity, Critical policy analysis

## Abstract

**Background**: COVID-19 had a major global impact on education, prompting concerns about its unequal effects and some impetus to reboot equity strategies. Yet, policy processes exhibit major gaps between expectations and outcomes, and inequalities endured for decades before the pandemic. Our objective is to establish, from education research, how policymakers seek equitable outcomes. Our study emulates its partner review of ‘Health in All Policies’ (HiAP) to ask: How does education equity research use policy theory to understand policymaking?

**Methods**: A qualitative systematic review (from 2020-21), to identify peer reviewed research and commentary articles on education, equity, and policymaking, in specialist and general databases (ERIC, Web of Science, Scopus, Cochrane/ Social Systems Evidence). We did not apply additional quality measures. We used an inductive approach to identify key themes. We use these texts to produce a general narrative and explore how relatively theory-informed articles enhance it.

**Results**: 140 texts (109 articles included; 31 texts snowballed) provide a non-trivial reference to policymaking. Limiting inclusion to English-language produced a bias towards Global North articles. The comparison with HIAP highlights distinctive elements of education research. First, educational equity is ambiguous and contested, with no settled global definition or agenda (although countries like the US, and organisations like the World Bank, have disproportionate influence). Second, researchers critique the narrow equity definitions – focusing on performance – that dominate policymaking. Third, more studies provide ‘bottom-up’ analysis of ‘implementation gaps’. Fourth, more studies relate inequity to ineffective policymaking to address marginalised groups.

**Conclusions**: Few studies use policy theories to explain policymaking, but there is an education-specific literature performing a similar task. Compared to HIAP research, there is more use of critical policy analysis to reflect on power and less focus on delivering top-down aims. Most studies criticise current educational equity aims and expect them to fail.

## Plain language summary

Governments and international organisations have made a longstanding commitment to education equity. Rebooted initiatives to incorporate the additional unequal impact of COVID-19 are possible, but policymaking research highlights likely obstacles to their progress.

First, equity is vague and there are many competing ‘education equity’ initiatives. International agendas focus on: shifting resources towards early years education; delivering a minimum level of schooling and making school environments more inclusive, to address the links between social and economic background (including class, gender, race, and ethnicity) and attainment; comparing the performance of school systems, including their ability to reduce inequalities of attainment; and, widening access to further and higher education (FE/HE).

Second, there is a continuous gap between expectations and outcomes. A ‘top down’ perspective, through the lens of international organisations or central governments, highlights implementation gaps. A ‘bottom up’ perspective, through the lens of local or school leaders, highlights an inability to make progress without understanding how people make sense of equity as they deliver policy.

Third, many possible outcomes can emerge in a complex policymaking system. The competition to define equity produces different agendas competing for resources. The ‘neoliberal’ performance management agenda narrows equity to a measure of school access or exam outcomes, while ‘social justice’ approaches address underlying causes of inequalities. ‘Equity for all’ approaches undermine attention to marginalised groups. Further, international, national, and subnational policymakers make sense of these agendas in different ways, and there is some ability for local policymakers to reinterpret central government initiatives. 

Overall, educational equity policymaking involves the exercise of power to decide what equity means, who matters, how to deliver policy, and who benefits. A technical focus on rebooting initiatives and closing implementation gaps does not guarantee success and overshadows the need to address wider determinants of education outcomes.

## Introduction

We present a qualitative systematic review of education equity policy research. The review describes the contested nature and slow progress of education equity agendas, how education research tries to explain it, and how the use of policy process research might help. The reviewed research was published before the global pandemic. However, the impact of COVID-19 is impossible to ignore because it has highlighted and exacerbated education inequities (defined simply as unfair inequalities). New sources include the unequal impact of ‘lockdown’ measures on physical and virtual access to education services (from pre-primary to higher education), often exacerbated by hastily rewritten rules on examinations ( Kippin & Cairney, 2021). The COVID-19 response has also highlighted the wider socio-economic context where some populations have the ability to live and learn *safely* while others do not.

This new international experience *could* prompt a major reboot of global and domestic education equity initiatives. It is *tempting* to assume that high global attention to inequalities will produce a ‘window of opportunity’ for education equity initiatives. However, policymaking research warns against the assumption that major and positive policy change is likely. Further, equity policy research shows that policy processes contribute to a major gap between vague expectations and actual practices and outcomes ( Cairney & St Denny, 2020). Crises could prompt policy choices that *exacerbate* the problem. Indeed, the experience of health equity policy is that the COVID-19 response actually *undermined* a long-term focus on the social and economic causes of inequalities ( Cairney *et al*., 2021).

Therefore, advocates and researchers of education policy reforms need to use policy theories to understand the processes that constrain or facilitate equity-focused initiatives. Without this understanding, equity policy research may tell an incomplete story of limited progress and address ineffectively the problem it seeks to solve. In that context, the guiding question of our review is: *How does education equity research use policy theory to understand policymaking?*

We designed this study as a partner to the review of the international health equity strategy *Health in All Policies* (HiAP) ( Cairney *et al*., 2021) to produce reviews of equity research in different policy sectors. The pursuit of equitable policy is impossible to contain within one sector, and comparison is crucial to our understanding of intersectoral policymaking (explored in Cairney *et al*., 2022). We did not expect to replicate the HiAP study entirely, since – for example - the terminology to describe policy aims and processes is not consistent across sectors, and the most-studied countries differ in each sector. Rather, we emulate the method for searching for articles: using *broadly* comparable search terms, while recognising that there is no direct education equivalent to HiAP; and, using the same broad focus on policy theories to guide inclusion. Then, we highlight key differences to structure our analysis. The health/ education equity comparison prompts us to:

***1. Establish if there is a coherent international education policy agenda to which each article contributes.***

The HiAP story is relatively coherent and self-contained, identifying the World Health Organization (WHO) ‘starter’s kit’ and notional playbook. HiAP research tends to support that agenda ( Cairney *et al*., 2021). In education, initiatives led by the United Nations Educational, Scientific, and Cultural Organization (UNESCO) have some comparable elements. However, there are (1) more international players with high influence, including key funders such as the World Bank and agenda setters such as the Organisation for Economic Co-operation and Development (OECD), and (2) more important reference points for domestic studies. In particular, US studies are relatively self-contained - examining the connection between federal, state, and local programmes – *and* the US model of education equity is a reference point for international studies.

***2. Analyse the contested definition of equity: what exactly does it mean?***

In politics, actors exercise power to resolve *policy ambiguity*: to determine who is responsible for problem definition and who benefits from their definition. The HiAP story contains the same basic treatment of equity as the avoidance of unfair health inequalities caused by ‘social determinants’ such as unequal incomes and wealth, access to high quality education, secure and well-paid jobs, good housing, and safe environments. This approach is part of a political project to challenge a focus on individual lifestyles and healthcare services. Few HiAP studies interrogate this meaning of equity before identifying a moral imperative to pursue it, although most find that policymakers do not share their views ( Cairney *et al*., 2021). In education, contestation is a central feature of research: equity is relatively vague and contested, there is no equivalent agreement that all inequalities are unfair, and fewer studies examine the ‘social determinants’ of education inequalities. Far more studies criticise how policymakers defend a more limited definition of equity as the equal opportunity to access a high-quality public service (the meaning of terms such as ‘quality’ are also contested – see Ozga *et al*., 2011).

***3. Explore critiques of ‘neoliberal’ approaches to education equity.***

Common descriptions of neoliberalism refer to two related factors:

1. Policymaking based on a way of thinking that favours individualism and non-state solutions, and therefore prioritises individual over communal or state responsibility, market over state action, and/or quasi-markets for public services (a competition to deliver services, designed and regulated by governments). For example, Rizvi (2016: 5) describes ‘a mode of thinking that disseminates market values and metrics to every sphere of life and constructs human beings and relations largely in economic terms’. A neoliberal approach to education equity would emphasise individual student motivation, quasi-market incentives such as school vouchers, and limited state spending in favour of private for-profit provision.
2. Giving relative priority to policies to ensure economic growth, with education treated as facilitating a ‘global knowledge economy’ rather than a wider social purpose ( Rizvi & Lingard, 2010: 39–41; Wiseman & Davidson, 2021: 2–3).

The damaging effect of neoliberal approaches – including their highly unequal effects within and across countries - is a central theme in health and education research. Half of all included health studies describe experiences - in Australia and Nordic countries - of high HiAP commitment undermined by a neoliberal economic agenda. Two-fifths of education articles focus on the United States and more describe the US as an international reference point. US education equity policy supports a model built on closing an ‘achievement gap’ via quasi-markets, quality improvement, performance management, and measuring the gap narrowly with standardised test scores. The US contributes disproportionately (alongside international organisations like the World Bank) to a limited focus on social determinants in favour of seeing education as an investment in human capital. While health studies analyse neoliberalism as an external disruptor to HiAP, education research centres and problematises it, to understand its tendency to constrain the equity efforts of national and local policy actors.

***4. Compare top-down and bottom-up perspectives on policymaking complexity.***

HiAP has a top-down focus, identifying the extent to which a policy agenda is implemented in different contexts. Few studies focus on health services, assuming that the biggest determinants of health are outside of healthcare. Education studies have a relatively bottom-up focus, identifying a national policy agenda as key context, but also local venues where actors make policy as they deliver. There is a greater focus on ‘sense making’ among school leaders.

***5. Identify the impact of minoritization and marginalisation.***

Education studies are more likely to centre race and racism, often using ‘critical policy analysis’ (research to defend marginalised populations when analysing policy problems and proposing solutions). These issues are not *absent* in HiAP research ( Baum *et al*., 2019; Bliss *et al*., 2016; Corburn *et al*., 2014; see also D’Ambruoso *et al*., 2021; Selvarajah *et al*., 2020). However, the included education studies have a greater focus on minoritization (the social construction of minority groups, and the rules to treat them in a different way from a dominant majority) and the equity initiatives that – intentionally and unintentionally - fail to address race and racism.

Our Discussion section relates this research to policymaking concepts and theories. The results of the review map onto well-established themes in policymaking research: policy ambiguity and contestation to define the policy problem; policymaking complexity and ‘emergence’ during implementation; policy transfer and convergence; and, the connection between ‘rationalist’ policy analysis, policy theories, and critical policy analysis.

## Methods

Our general aim is to identify how policymakers and researchers frame the policy problem of equity, fairness, or justice in relation to inequalities (for example in relation to geography, gender, class, race, ethnicity, and disability). We also examine how they relate policy solutions to this framing. Our general focus in the *Integrative Mechanisms for Addressing Spatial Justice and Territorial Inequalities in Europe* (IMAJINE) project is:

1. **What is the policy problem?** Specifically, what is equity, and what constrains or facilitates its progress?
2. **How does it relate to policy processes?** Do articles identify a lack of policy progress and how to address it? What theories do they use when describing policymaking?

In that context, our review’s guiding question is: *How does education equity research use policy theory to understand policymaking?*

Originally, we identified five sub-questions:

1. How many studies provide a non-trivial reference to policymaking concepts or theories?
2. How do these studies describe policymaking?
3. How do these studies describe the ‘mechanisms’ of policy change that are vital to equity strategies (although Cairney *et al*., 2021 show that very few studies answer this question)?
4. What transferable lessons do these studies provide? For example, what lessons for other governments do case studies provide?
5. How do these studies relate educational equity to concepts such as spatial justice? (we answer question 5 in Cairney *et al*., 2022).

As Cairney *et al*. (2021) describe, our reviews set a lower bar for inclusion than comparable studies, based on previous work which shows that a wide search parameter and low inclusion bar (in relation to relevance, not quality) does not produce an unmanageable number of articles to read fully. Further, high inclusion fosters ‘immersion’ and helps us to generate a broad narrative of the field, identify the most policy theory-informed articles, and examine how these articles enhance that narrative.

We searched fewer databases than Cairney *et al*. (2021), partly to favour snowballing to generate core references identified by authors of included articles. We also searched each database sequentially to use feedback from each search to refine the next and pursue a sense of ‘saturation’. Initially, we used the education-specific database Institute of Education Services (ERIC) in 2020 (search ran from 18/10/20 to 20/12/20). We used these search terms: ‘education’, ‘equity’, and ‘policy’, with no additional filters, then sought articles providing one or more references to (a) the ‘policy cycle’ (or a particular stage, such as agenda setting or implementation) or (b) an established *policy theory*, such as multiple streams, the advocacy coalition framework, punctuated equilibrium theory, or *concept*, such as variants of new institutionalism (we used Cairney, 2020 for a list of theories and concepts, summarised on Cairney’s blog). These are broadly comparable search terms, but there is no direct equivalent to HiAP or the WHO as its champion. UNESCO is broadly equivalent to the WHO, but to focus on a UNESCO initiative alone would be misleading: the WHO features in almost-all HiAP studies, but UNESCO is discussed less frequently than the World Bank or OECD in education studies.

We used similar search criteria for inclusion as Cairney *et al*. (2021). The article had to: be published in a peer reviewed journal in English (research and commentary articles), and provide at least one reference to a conceptual study of policymaking in its bibliography (including articles that cite education policymaking texts rather than the original policy theory source). This focus on articles alone seems more problematic in education, so we used snowballing to identify 31 exemplar texts described as foundational. Education research has its own frames of reference regarding: ‘policy sociology’ (half of the included articles feature Ball, e.g., Ball, 1993; Ball, 1998; Ball *et al*., 2011), policy borrowing (e.g., Rizvi & Lingard, 2010; Steiner-Khamsi, 2006; Steiner-Khamsi, 2012), policy implementation (e.g., Spillane *et al*., 2002), and performance management (e.g., Ozga *et al*., 2011). Most articles describe concepts such as policy transfer without relying on the ‘mainstream’ policy theory literature ( Cairney, 2020), but, for example, Rizvi & Lingard (2010) and Steiner-Khamsi (2012) perform this function.

**Figure 1.**
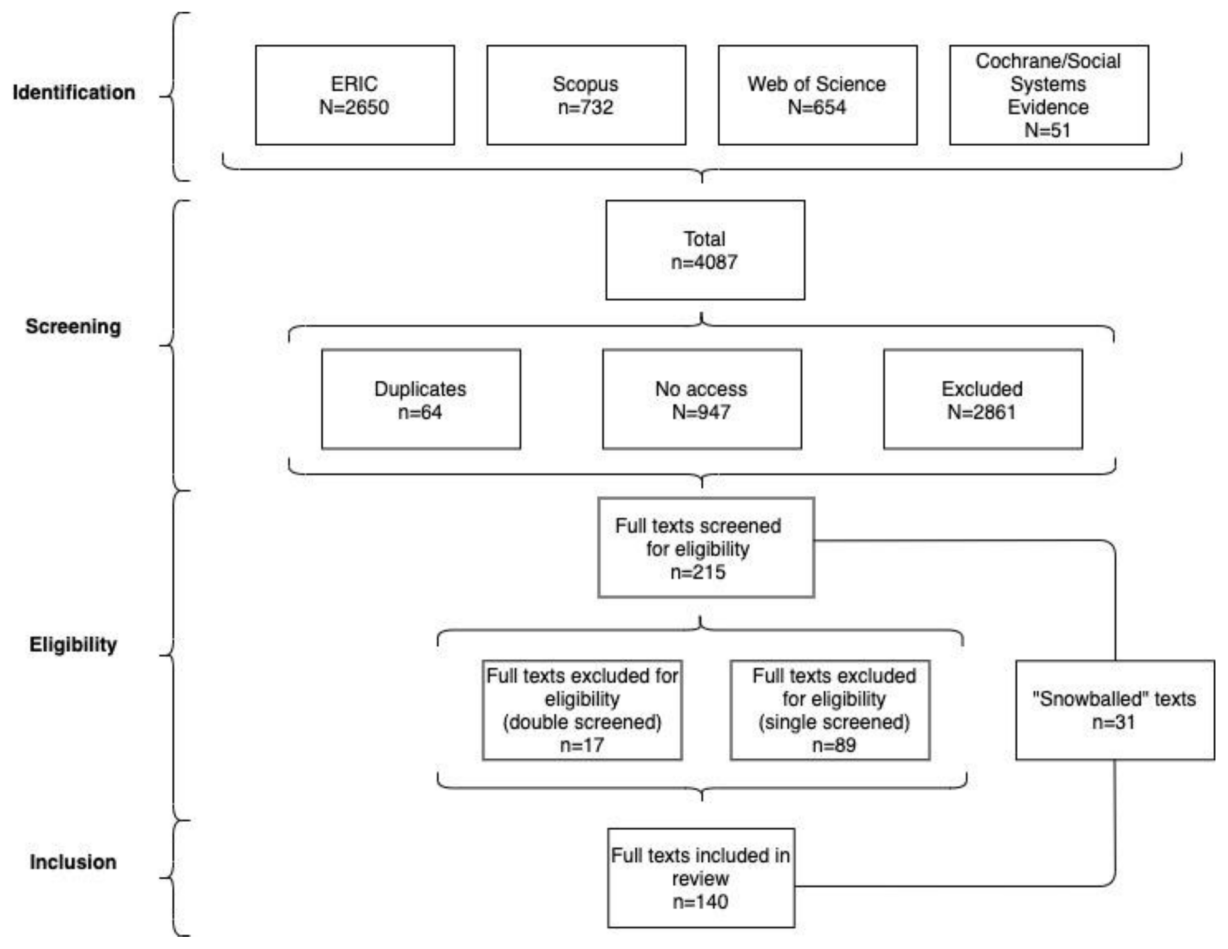
Review process flow chart.

This initial approach to ERIC – inclusion, immersion, snowballing – allowed us to establish the limited relevance of articles with a trivial reference to policy concepts. We could then pursue a more restrictive approach to subsequent searches: using the same search terms (education*, equit*, policymak*) and no additional filters, but erring towards manual exclusion when the article had a minimal discussion of policymaking. Searches of Cochrane/ Social Systems Evidence database (01/06/21 – 02/06/21), Scopus (29/03/21 – 23/04/21), and Web of Science (05/05/21 – 27/05/21), found 26 additional texts before we reached saturation. Table 1 and Figure 1 summarise these search results.

**Table 1.** Search results 2020/21.

Database	Search results	Duplicates	No access	Excluded at any stage	Included
**ERIC**	2650	2	519	2046	83
**Scopus**	732	21	215	483	13
**Web of Science**	654	41	213	390	10
**Cochrane/** **Social Systems Evidence**	51	0	0	48	3
**Total**	4087	64	947	2967	109

The search for articles is never comprehensive, and we identify the following risks of bias among the selected studies. We did not restrict by geography *directly*, but our exclusion on the basis of language (English) and initial use of a US database influenced geographical coverage. Most studies are of Global North countries and the US in particular. We did not exclude on the basis of quality, but 79% of included articles drew on new research (below).

Kippin carried out the initial ERIC screening, producing a long list - erring on the side of inclusion - based on the title, abstract, bibliographies, and a brief search to check for the non-trivial use of ‘policymaking’ in the main body of the text. Cairney performed a further inclusion check on the long list, based on a full reading of the article (to extract data as part of the review), referring some articles back to double check for exclusion. Cairney and Kippin double-screened 17 borderline cases during the final eligibility phase (using full-text analysis). In this stage, we excluded 10 borderline cases but included seven that provided a comparable study of policymaking without citing mainstream policy theories. In total, 83 articles are included from ERIC ( Figure 1). The same process yielded three articles (one excluded) from Cochrane/ Social Systems Evidence databases, 13 (two) from Scopus, and 10 (two) from Web of Science.

We extracted from each included article (total: 109): the definition of educational equity, the ‘story’ of the article (a summary of its key messages, and recommendations if provided), the use of policymaking concepts, as well as:

- Country/region of study. 43% of studies focused on the US, 9% Canada, 8% Nordic countries, and 7% Australia. 15% described multi-country studies.
- Country of author affiliation. 50% of first authors were listed as affiliated with organisations in the US, 14% Canada, 14% Australia, and 7% Nordic countries.
- Policy or case study issue. Nearly all described compulsory primary and/or secondary education (91%) or Higher Education (HE) (6%). 3% were ‘other’ (e.g., vocational education or system-wide studies).
- Research methods. Studies used semi-structured interviews with policy participants (28%), document analysis (16%), surveys and statistical analysis (8%), discourse/narrative analysis (7%), ‘systematic’ or ‘rigorous’ reviews (5%), case study methods (5%), content analysis (4%), participant observation (4%) or other methods (2%). 21% did not describe a research method.
- Article type. Included texts were research articles or reviews (83%) or commentary articles (17%).

We used an inductive qualitative approach to identify key themes – including the contestation to define education equity, and the uneasy balance between centralised and decentralised approaches to policymaking - from each paper, using immersion to understand how education research conceptualised policymaking. Compared to HiAP, we found (a) a greater focus on critical policy analysis to problematise how policymakers define problems and seek solutions, (b) almost no equivalent to the instrumental use of policy theories (except Eng, 2016), and (c) a greater willingness and ability to understand policy processes.

The complete search protocol is stored on the OSF ( https://doi.org/10.17605/OSF.IO/BYN98) ( Cairney & Kippin, 2021).

## Results

### The policymaking context: how international organisations frame equity

Education equity policy is heavily contested, producing multiple competing agendas. Yet, most articles identify a tendency for one approach to dominate in relation to (1) global equity initiatives (such as to boost school capacity) and (2) the impact of international agendas on domestic policy. In that context, we first describe the wider international context in which most articles are situated. Throughout, we use a comparison with HiAP ( Cairney *et al*., 2021) to note the relative absence of a single global and domestic equity agenda in education policy and research.

### Global equity initiatives

On the one hand, as with HiAP, there is a well-established global agenda championed by an UN organisation. UNESCO’s approach to educational equity is often similar to the WHO approach to HiAP (see Cairney *et al*., 2021). Broad comparable aims include:

- Treat education as a human right, backed by support for countries to produce legal and political obligations ( UNESCO, 2021b).
- Foster inclusion and challenge marginalisation ‘on the basis of socially-ascribed or perceived differences, such as by sex, ethnic/social origin, language, religion, nationality, economic condition, ability’ ( UNESCO, 2021a).
- Foster gender equality, to address major gaps in access to education ( UNESCO, 2021c).
- Boost early education (0–8 years) as the biggest influence on human development and most useful investment ( Marope & Kaga, 2015; UNESCO, 2021d).
- Boost the mutually reinforcing effect of education and health ( UNESCO, 2021e).
- Boost global capacity, such as via teacher recruitment ( UNESCO, 2021f).
- ‘Ensure inclusive and equitable quality education and promote lifelong learning opportunities for all’ (UN Sustainable Development Goal 4, SDG4).

On the other hand, there are competing narratives on what equity means in this context, including:

1. *The primary purpose of education*: as training for work, as part of an economic ‘human capital’ narrative (supported by ‘donor’ organisations such as the World Bank, and country government organisations such as United States Agency for International Development, USAID); or to foster student emancipation, wellbeing, and life opportunities (supported by education researchers and practitioners) ( Faul, 2014; Vongalis-Macrow, 2010).
2. *The meaning of ‘education for all’*: shifting since 1990 to treating education solely as schooling (and prioritising targets for primary schools), and changing the meaning of ‘for all’, “from encompassing all countries to developing countries only; from ‘all’ to children only; and from being a responsibility of all members of the international community to being a responsibility of governments to their citizens alone” ( Faul, 2014: 13–14; Gozali *et al*., 2017: 36).
3. *Narratives of inclusion*: including the UNESCO Salamanca statement on inclusive special needs education, global commitments to education for girls, and some focus on the ‘social determinants’ of learning related to class, race, ethnicity and marginalisation, or the need for multicultural education to challenge racism and xenophobia ( Engsig & Johnstone, 2015; Faul, 2014: 15; Lopez, 2017).
4. *Narratives of high ‘quality’ education*: including a tendency to focus on reading and mathematics, with limited support for ‘the role of education in broad social issues and its intrinsic value’ ( Faul, 2014: 16).
5. *Who should deliver education*: the public or private provision of services.

As Faul (2014: 16) describes, approaches to these questions fall into two broad categories: 

*1. An economic approach supported by performance management* (fostered by the World Bank, International Monetary Fund, and countries such as the US). It measures learning in relation to test-measured outcomes, facilitated by techniques associated with new public management (NPM), privatization and the mantra of ‘evidence based’ policy. Klees & Qargha (2014: 324–5) argue that this ‘cheap fix’ approach exacerbates inequalities while pretending to reduce them. Further, the analysis of results are highly contested in areas such as ‘performance pay for teachers, low-cost private schools, teacher training, conditional cash transfers, and most other studies of impact’ (2014: 329; see also Tobin *et al*., 2016: 583 on large scale assessments ‘to diagnose issues and target interventions for identified disadvantaged groups’).

2. *A human rights and social determinants approach* (fostered by UNICEF and UNESCO). A ‘rights-based, social justice argument calls for universal investment in quality education regardless of its impact’ ( Klees & Qargha, 2014: 330). UNICEF supports an approach that describes ‘deeply entrenched structural inequalities and disparities’ which keep ‘children out of school’, albeit often vaguely, and while diluting their language by also referring to cost-effectiveness measures of success (2014: 326–7; 330–1).

The former approach dominates international policymaking, prioritising literacy and numeracy, and measuring access in narrow ways (e.g., ‘gender parity’ as ‘equal numbers of boys and girls in school’, 2014: 17). The latter receives rhetorical support without being backed by concrete measures (and UNESCO policy statements generally come with descriptions of limited progress). There is also a tendency towards technocracy, with limited democratic and participatory processes to help define policy ( Klees & Qargha, 2014: 331).

Consequently, narratives of long-term development describe progress in global education (a shift over 60 years from a global minority to majority receiving some kind of education), but unequal progress, with a warning against one-size-fits-all approaches to access ( Reimers *et al*., 2012). Klees & Qargha (2014: 321–3) identify a gap between global rhetoric and actual practices regarding Education for All (EFA, which preceded SDG4). The Universal Primary Education (UPE) commitment has existed since the 1960s, but there is no prospect of the equivalent for secondary education (2014: 322), suggesting that: ‘these efforts have not been sufficiently serious’ (2014: 325–6). The gap relates partly to the alleged trade-offs, such as with efficiency or quality, that undermine support for equity (2014: 324). There are also many international organisation initiatives (including USAID on reading skills; World Bank Learning for All, Brookings Institution Global Compact for Learning) and initiatives funded by corporate or philanthropic bodies, each with their own definitions, motivations, and measures ( Tarlau & Moeller, 2020).

This story infuses most comparative studies. For example, Vaughan’s (2019: 494–6) discussion of financial support for gender-based education equity identifies shifts in focus, including: on women’s rights (up to 1990), equal access to schools (1990–2010), and ‘gender-based violence’ and other social factors that undermine equality (a patchy focus since 2010). A rise in attention has generated new opportunities for women’s rights groups and social movements to influence policy (2019: 500–8), but has not prompted a shift from the dominant economic frames of equity supported by ‘multilaterals, bilateral agencies, national governments and more recently, private sector organisations’ (2019: 494). These organisations measure ‘gender disparities in access, attendance, completion and achievement’, drawing ‘heavily on human capital perspectives concerned with the economic significance of getting girls into school, particularly in terms of poverty reduction’ (2019: 509; 496). This focus on a ‘business case’ for policy minimises attention to the marginalisation of girls within schools and the need to reform systems to ‘properly change how schooling relates to gender inequalities in the labour market, political participation, and levels of violence against women’ (2019: 509–10; 496).

Literature reviews - commissioned by development agencies on ‘developing countries’ - also identify patchy evidence and limited progress ( Kingdon *et al*., 2014 and Novelli *et al*., 2014 are for the UK Department for International Development; Best *et al*., 2013 is for Australian Agency for International Development). Kingdon *et al*.’s (2014) ‘rigorous review of the political economy of education systems in developing countries’ finds that the putative benefits of (neoliberal international donor-driven) education decentralisation ‘do not accrue in practice’, particularly in rural areas (in e.g., Mozambique, Zambia, Zimbabwe, Mexico, Indonesia, Ghana) (2014: 2; 28–9). Best *et al*. (2013: 65) find that ‘Almost two-thirds of all developing countries have participated in a national, regional or international assessment programme’, but find minimal evidence of their impact. Novelli *et al.* (2014: 40–2) describe the amplification of problems in ‘conflict affected contexts’, where security actors overshadow humanitarian actors and education specialists are marginalised. In that context, global agendas on access to school have a ‘one size fits all’ feel (e.g., Nepal), the prioritisation of post-conflict economic growth and education efficiency/ decentralization often exacerbates material and educational inequalities (e.g., El Salvador, Guatemala, Nicaragua, Honduras), a focus on equity in relation to citizenship often distracts from inequitable allocations of resources (e.g., Sri Lanka), and the insistence on free primary education obliges large private sector expansion (e.g., Rwanda).

### International agendas on equity, performance, and quality in education

Many organisations seek to measure and promote improved performance in education systems and schools as the main vehicle for equity. The OECD is particularly influential ( Grek, 2009: 24; Grek, 2020; Rizvi & Lingard, 2010: 128–36). It has a wide remit, engaging with multiple definitions of equity and ways to achieve it, despite being associated with a focus on education system performance management via international testing programmes such as PISA (Programme for International Student Assessment). Key reports describe education equity in relation to human rights and socio-economic factors; education is a basic necessity that boosts health, wellbeing, citizenship, and economies ( Field *et al*., 2007: 11; 33; OECD, 2008: 1). The OECD ( OECD (2008); OECD (2012); ( Field *et al*., 2007: 11; 31, drawing on Levin, 2003) relates equity to:

1. *Fairness* (social background should not obstruct education potential), *inclusion* (everyone should reach a minimum standard), and *opportunity* (to receive education and succeed at school) ( OECD, 2008: 2).
2. *The imperative to address unfair inequalities.* There remains a gap between ambitions and outcomes, and major inequalities of attainment endure in relation to poverty, migration, and minoritization ( Field *et al*., 2007: 3; OECD, 2008: 2).
3. *Costs.* Inequalities have individual costs (relating to income, citizenship, and the ability to learn) and social costs (including economic stagnation and public service costs) ( OECD, 2012: 3; Field *et al*., 2007: 33).

It also sets international policy agendas, identifying the ability of (a) *good school performance*, and (b) the *distribution of education spending* (in favour of early years over higher education) to mitigate against socio-economic inequality ( Field *et al*., 2007: 22; 39; OECD, 2012: 9; 3; OECD, 2008: 2; 6–7; OECD, 2015: 1–2).

Overall, the OECD relates inequitable outcomes to ‘deprived backgrounds’ and ‘weak schooling’ ( Field *et al*., 2007: 26). It recognises the ‘lack of fairness’ caused by the unequal impact of ‘socio-economic background’ on school completion and attainment (2012: 9), and has *some* HiAP-style emphasis on cross-sectoral working and supportive social security: ‘education policies need to be aligned with other government policies, such as housing or welfare, to ensure student success’ (2012: 10). However, it does not share with HiAP the sense that all unequal outcomes are unfair and require state intervention, since some relate to individual motivation and potential ( Levin, 2003: 5, cited in Field *et al*., 2007: 31). Levin (2003: 8) describes a balance between ‘equality of opportunity’ and equitable outcomes in skills attainment and employability. Nor do they support the HiAP focus on ‘upstream’ whole-population measures ( Cairney *et al*., 2021). Rather, equity is the fair distribution of good education services, on the expectation that education can largely solve inequities relating to a minimum threshold of attainment ( Field *et al*., 2007: 26). This focus on ‘helping those at the bottom move up’ is ‘workable from the standpoint of policy’ ( Field *et al*., 2007: 31; 46–51; Levin, 2003: 5).

In that context, the OECD makes the following recommendations:

1. *Foster the equitable distribution of budgets.* Prioritise funding for high quality early education, free or reduced-fee education, and reducing regional disparities ( Field *et al*., 2007: 23; 122–6; OECD, 2012: 3–11; 117–8; OECD, 2008: 5; OECD, 2015).
2. *Foster multiculturalism and antiracism*. Foster a ‘multicultural curriculum’ and improve support such as ‘language training’ for immigrant students ( Field *et al*., 2007: 150–1; OECD, 2008: 2). Challenge the disproportionate streaming of ‘minority groups’ into special education (2007: 20).
3. *Reform school practices.* Make evidence-informed choices to address equity and ‘avoid system level policies conducive to school and student failure’ ( OECD, 2012: 10).

- Repeating a school year is ineffective and exacerbates inequalities ( Field *et al*., 2007: 16–18; OECD, 2008: 4–5).
- *Tracking*. Early tracking and selection (assigning students to different classes based on actual or expected attainment) exacerbates inequalities without improving overall performance (2008: 4; 2012: 11). Poor selection practices reduce the quality of education and ‘peer-group’ effects, increase stigma, and are based on unreliable indicators of future potential ( Field *et al*., 2007: 59).
- *School choice*. Parental choice on where to send their children can exacerbate inequalities related to demand (e.g., some have more resources to gather information and to pay for transport) and supply (e.g., the discriminatory rules for entry) (2008: 3; Field *et al*., 2007: 15; 62–4; see also Heilbronn, 2016).

4. *Seek effective school governance to* ‘ *help disadvantaged schools and students improve*’ ( OECD, 2012: 11). Develop capacity in school leadership, provide ‘adequate financial and career incentives to attract and retain high quality teachers in disadvantaged schools’ (2012: 12), reject the idea that ‘disadvantaged schools and students’ should have lower expectations for attainment (2012: 12), and take more care to foster links with parents and communities to address unequal parental participation (2012: 12). ( Field *et al*., 2007: 19; OECD, 2012: 11–12; OECD, 2008: 5).
5. *Avoid the inequitable consequences of performance management and league tables.* Measurement and targets can be useful to identify (a) unequal early-dropout rates and rates of attainment at school leaving age, and (b) school performance in reducing inequalities ( OECD, 2008: 7). However, the publication of crude league tables of good versus bad schools exacerbates uninformed debate (2008: 7; Field *et al*., 2007: 131).

### The overall international context for our review of education equity policy

While UNESCO is not *absent* from our review, the majority of articles identified in this review are country studies that engage with reference points associated with the World Bank (neoliberal policy and policymaking) and OECD (performance management). Governments tend to describe reforms to improve equity via (a) access to higher quality schooling and (b) reaching a minimum attainment threshold on leaving school. At the same time, they respond to the pressures associated with international league tables that compare performance by country and compare school performance within each country (using measures such as PISA, Trends in International Mathematics and Science Study (TIMSS) and Progress in International Reading Literacy Study (PIRLS) – Grek, 2009: 27; Schuelka, 2013).

Consequently, equity policies focusing on social determinants, social justice, and inclusion, struggle to compete. They are overshadowed by more politically salient debates on the relationship between economic growth/ competitiveness and education, including the idea that we can quantify the relative performance of each country’s education system and use the data to improve each system ( Grek, 2009: 27; Rizvi & Lingard, 2010: 133–6). Almost all of these policies shelter under the umbrella term ‘education equity’ even if they achieve no such thing.

## Policy ambiguity: the competition to define and deliver equity

The included articles discuss a wide range of equity-related issues, in relation to: mixed-sex schools ( Zufiaurre *et al*., 2010), the proportion of girls in education or work ( Ham *et al*., 2011; Yazan, 2014), the representativeness of school leaders or parental involvement in relation to ‘race, gender, ethnicity, and social class’ ( Bertrand *et al*., 2018; Marshall & McCarthy, 2002: 498; Porras, 2019), language training for immigrant populations ( Brezicha & Hopkins, 2016: 367; Hara, 2017), the inclusion of the ‘Roma minority in Europe’ ( Alexiadou, 2019: 422), the fairness of teacher grading ( Novak & Carlbaum, 2017), school behavioural and expulsion measures ( Welsh & Little, 2018), access to health and physical education ( Penney, 2017), challenges to sex discrimination ( Meyer *et al*., 2018) or heteronormative schooling ( Leonardi, 2017), and encouraging equal access to vocational, further and higher education in relation to race, gender, socio-economic status or spatial justice, such as by developing regional college provision ( Gill & Tranter, 2014: 279; Pinheiro *et al*., 2016) or encouraging ‘student voice’ ( Angus *et al*., 2013). They describe initiatives that focus narrowly on school access and teaching ‘quality’ ( DeBray *et al*., 2014; Donaldson *et al*., 2016; Hanna & Gimbert, 2011; Louis *et al*., 2008), human rights to preschool education ( Mtahabwa, 2010), or the distribution of scarce resources ( De Lisle, 2012; Spreen & Vally, 2010).

However, most articles contribute to two themes. The first is the distinction between equity as ‘horizontal’ (treat equally-resourced people equally) or ‘vertical’ (treat unequally-resourced people unequally) in relation to access to opportunities, processes, or outcomes ( Gilead, 2019: 439; Rodriguez, 2004). Policy actors identify how reasonable it is for the state to intervene directly, or foster individual motivation backed by market driven measures to drive up school quality. Gilead (2019: 439) compares three common ways to describe equitable resource allocation, noting that the first two seem inadequate while the third receives inadequate support:

1. *‘Merit’*. A sole focus on individual effort produces ‘severe inequalities and a neglect of the weakest members of society’.
2. *Thresholds*. A focus on ‘improving the conditions of the least advantaged members of society’ such as via an attainment threshold, is feasible but allows ‘the stronger members of society to preserve their relative advantages’.
3. *Justice*. Options to ‘equate justice with equality’ include equal (a) *receipt of resources* (such as to reduce geographical inequalities), (b) *opportunities to education* (although the meaning of ‘opportunity’ is contested), and (c) *outcomes* (embraced rarely because ‘it advances an unrealistic and potentially socially harming ideal’).

Second, they link these contested definitions of equity to governance, prompting most researchers to ask: (1) whose definition of equity matters, (2) what means to achieve equity do they prefer, and (3) who should be responsible for equitable opportunities and outcomes? Most articles situate these discussions in relation to dominant, narrow definitions of school-driven equity, generally to highlight their limitations. They describe policymakers using the *word* ‘equity’ without establishing a clear mechanism to secure it, in a multi-level policymaking system over which they have limited control.

For example, many central governments pursue equal access to schools: favouring distribution and regulation (funding and regulating schools) over redistribution (taxing high income to compensate low income populations), and holding schools responsible for variations in outcomes despite social inequalities that are not amendable to change by education sectors alone. Further, central governments do not define equity policy well, increasing the possibility that local actors (including district and school leaders) can change policy as they deliver. The overall result is often a tension between multiple definitions of equity pursued in multiple levels of government.

In that context, we relate the included studies to two main categories:

1. *Critiques of dominant definitions in international and domestic agendas*. This section describes the US as an exemplar of a problematic neoliberal model of equity policy, with most other countries presenting variations on the same theme.
2. *Competing definitions and alternative aspirations*, focusing on a well-regarded model (Finland), and the standards or values that researchers use to analyse real-world practices.

### 1. Critiques of dominant definitions in international and domestic agendas

***The US: ill-defined and contested equity.*** US studies treat equity as an often used but ill-defined and contested term. Ambiguity makes it difficult to clarify the implications for policy, and the intentional or unintentional lack of clarity exacerbates inequalities ( Bulkley, 2013; Chu, 2019). Contestation relates to horizontal versus vertical definitions:

‘Horizontal equity is concerned with providing equal treatment and provisions to all schools and students whereas vertical equity is concerned with ensuring that students with greatest needs or in disadvantaged conditions will receive more resources … The horizontal perspective of equity is similar to … a “thin” equity that prioritizes individuals’ equal access to educational resources and opportunities. In contrast, a “strong” equity recognizes the historical, socioeconomic, and racial inequities in education and calls for a structural, transformative approach to stop and uproot inequity’ ( Chu, 2019: 5, citing Cochran-Smith *et al*., 2017; see also Halverson & Plecki, 2015)

This distinction helps identify a spectrum of support for government intervention: simply ensuring procedural fairness in schools while assuming a meritocracy; redressing inequalities to encourage fairer competition; and, redistributing educational resources to ensure that no one dips below a performance threshold ( Bulkley, 2013: 11; Kornhaber *et al*., 2014). Bulkley’s (2013: 10) interviews of education researchers, advocates, and practitioners highlight disagreement on:

- *How to distribute inputs*: such as an equal ‘opportunity to learn’ in a classroom. Most seek more resources – including ‘high quality’ teachers - for students in (a) high poverty areas (b) attending schools with lower resources (teaching and technology), and (c) likely to interact with teachers with less experience and more turnover (2013: 15–16). One exception was the American Enterprise Institute which argues that redistribution would reduce overall quality and performance and disadvantage better performing middle class schools (2013: 16; 20).
- *How to set boundaries between education and other policy domains*: How to define ‘low income’ and set boundaries between public education and other policies with a major influence on learning (e.g., on health, nutrition, housing) (2013: 11). Some call for more recognition of the wider context; others think it lets schools off the hook for their performance (2013: 16).
- *Who should be responsible*, *and what they should they do*: Debates focus on reforming existing services or introducing more market mechanisms (2013: 17). They focus on course content, classroom practices, segregation by socioeconomic status, the governance of schools, the allocation of teacher time, and incentives such as school vouchers (2013: 12).
- *How to set expectations for equity of outcomes:* Debates on the appropriate outcomes in relation to attainment - ‘equity as equal outcomes, equity as meeting a threshold, and equity as making progress’ - include an adequate threshold to allow social, economic, and political participation, plus a judgement on how much equalization of achievement is possible or desirable ( Bulkley, 2013: 12; 18). Outcomes can refer to reducing gaps in attainment or the link between attainment and employment. Thresholds include graduating high school or being college ready.

One way to address this ambiguity is to exercise power – via professional discourse and political processes - to resolve contestation in favour of one definition. However, Chu (2019: 3) finds that state governments define equity vaguely. There is *some* government action to set expectations, but many are clarified in practice. This lack of care to define a social justice-oriented agenda minimises the challenge to individualist notions of education built on neoliberalism, market mechanisms, and performance management ( Bishop & Noguera, 2019; Evans, 2009; Hemmer *et al*., 2013; Horn, 2018; Lenhoff, 2020; Trujillo, 2012; Turner & Spain, 2020).

***Australia: equal access to schooling in an unequal socioeconomic and spatial context.*** Australian studies critique a tendency to connect (a) giving ‘everyone a chance at the same outcomes’ regardless of wealth or culture, to (b) access to schooling, rather than (c) the social determinants of unequal outcomes ( Loughland & Thompson, 2015; Taylor, 2004: 440). The wider context is a highly stratified society exacerbated by private versus public education: disadvantaged students go to state schools while others go to the better funded and performing private sector, with fee-paying schools also subsidised by the federal government ( Loughland & Sriprakash, 2016: 238; Morsy *et al*., 2014: 446). The education system is designed to encourage unequal outcomes via competition and performance management. Loughland & Sriprakash (2016: 238) describe a PISA-driven agenda which contributes to ‘a performative framework for equity’ conflating ‘quality and equity’ (2016: 238).

In other words, policymakers are *pretending* that the highest quality education is available to all ( Clarke, 2012: 184). Federal government descriptions of a ‘sector-blind’ policy, funding all schools, avoids discussing redistribution to address disparities in social background and achievement, while linking education to individual success and economic competitiveness rather than collective wellbeing ( Taylor, 2004). Morsy *et al*. (2014: 446) describe a strategy to depoliticise education equity to maintain existing inequalities of power and outcomes: (1) emphasising governmental neutrality, the technical aspects of policy, and the value of market mechanisms; (2) prioritising individual motivation, effort, and success; and (3) describing the welfare state as political and markets as natural. Overall, equity is about competition and performance, not social determinants or social inclusion (2016: 239–40). This approach exemplifies a wider international tendency to use performance measures and league tables to describe education inequalities as natural, fostering the ‘stigma of failure at institutional and individual levels’ that exacerbates wider social inequalities ( Power & Frandji, 2010: 394, describing England and France).

In that context, we can only make sense of the overall impact of equity agendas by relating them to the more supported policies that exacerbate inequalities in practice. In particular, Reid (2017) shows how the neglect of spatial injustice exacerbates the racial and ethnic inequalities that Australian governments seek to reduce: there is lack of access to high quality schooling in rural areas, which have relatively high Indigenous populations. There has been a “national emphasis since 2007 on ‘closing the gap’ in education, health and economic outcomes for Indigenous Australians”, with ‘education policy aimed at raising educational attainment by improving early education programs, preschool attendance, improving primary schooling, and providing financial incentives to attract experienced and successful teachers to the most disadvantaged schools’ (2017: 89). However, the wider policy context ‘operates to intensify and exacerbate the effects of dominant sociological issues of race, class, gender and geography’ (2017: 89; Molla & Gale, 2019).

Gill & Tranter (2014: 291) suggest that policymaker and media agendas exacerbate such problems by drawing incorrect conclusions from data. They describe the perception – derived wrongly from the rise of middle-class women going to university – that girls are more likely than boys to overcome class-based disadvantage. There is a long-term government and media concern about working class boys being marginalised in education - the ‘new’ disadvantaged in relation to ‘retention rates, expulsion and suspension rates, lower levels of literacy and social and cultural outcomes’ – without considering (say) their greater ability to receive the same employment opportunities with fewer qualifications ( Gill, 2005: 108–110; compare with Martino & Rezai-Rashti, 2013 on Canada). In contrast, gender equity movements focus on the often unspoken and unseen sources of inequity in relation to gendered roles in public and private, expectations in education and employment, and gendered violence ( Marshall, 2000).

***Canada: unequal outcomes masked by the rhetoric of equitable policies.*** Studies of federal and provincial policies in Canada highlight the lack of practical meaning of ‘equity policy’ rhetoric. Canada exemplifies a contrast between (1) the practices that exacerbate inequalities and (2) the vague rhetoric of equity that masks their effects. Practices include fiscal inequalities, in which unequal funding for schools from private funds relates inversely to need measured in socioeconomic terms ( Winton, 2018); some districts are relatively able to raise revenue in the market and use it to improve schools in that district (2009: 160). A general government commitment to equity of achievement – in relation to class, race, and gender – remains unconnected to finance and geography.

Further, equity policies related to anti-racism and multiculturalism (as opposed to assimilationism) are diluted by other agendas. George *et al*. (2020: 172–3) identify in British Columbia and Ontario a tendency for policy documents to describe individual rather than structural determinants of racism. Paquette (2001) describes failed bids in British Columbia to produce policies that reduce inequalities in relation to race, ethnicity, gender, and/or disability, in a context of (a) recession and limits to government spending and (b) a commitment to standardised testing to gauge individual and school performance. Segeren & Kutsyuruba (2012: 2) describe ‘a noticeable retrenchment with respect to equity policies’ in the Ontario Ministry of Education, with equity subsumed ‘under the banner of school safety, discipline, harassment, and bullying’ rather than anti-racism.

Authorities foster symbolic measures to look like they are addressing education inequity (largely in relation to a threshold of attainment) ( Hamlin & Davies, 2016). For example, Toronto’s global multicultural image helps mask important variations of experience (2016: 189). Further, Gulson & Webb (2013: 173) connect the ‘underachieving’ of black students in a Toronto district with many thwarted attempts to respond (over decades), such as proposals for a black-focused curriculum or to set up Afrocentric schools. The governance mechanisms exist to support this proposal, but it has faced disproportionately intense local opposition (2013: 171).

There is also a tendency towards rhetoric to address the transition to HE that exacerbates inequalities in education ‘on the basis of ethnicity, ability/disability, gender, sexuality, and religion’ ( Tamtik & Guenter, 2019: 41). There is a suite of potential approaches to inequalities, including: to foster inclusion, the value of difference, recognition, and a removal of barriers to education such as discrimination against students and cultural isolation; and, hiring and promoting staff from a wider pool of recruitment (2019: 43). However, most universities focus on minimum standards of attainment, while few relate fairness to redistributing resources.

***Multiple country studies: a general contrast between equality of access versus outcomes.*** Multiple country studies provide a similar contrast between dominant versus their preferred definitions of equity. They highlight a tendency to foster equality of access to education (often backed by market mechanisms such as voucher and school choice schemes) and measure outcomes narrowly, at the expense of a meaningful redistribution of resources or alternative measure of success:

- In Cyprus, a focus on access to schools, combined with limited school action, fails to address ‘the actual experience of marginalisation, disadvantage or discrimination’ and ‘points to cultural domination, non-recognition and disrespect’ ( Hajisoteriou & Angelides, 2014: 159).
- In Denmark, Engsig & Johnstone (2015: 472) identify the contradictions of educational ‘inclusion’ policies with two different aims: (1) social inclusion and student experience (the UNESCO model, adapting to students), and (2) mainstreaming in public education coupled with an increased focus on excellence and quality, via high stakes student testing to meet targets (the US model, requiring students to adapt).
- In Sweden and Norway, Pettersson *et al*. (2017: 724) describe the strategies used by policymakers to favour a neoliberal focus on equal access. In Sweden, it contributed to school choice agendas (including to foster independent school autonomy) that increased segregation ( Varjo *et al*., 2018: 482–3). A comparison with Finland suggests that such measures can still be highly regulated by central government (2018: 489–92).
- In Chile, advocates for markets argue that they increase access, for disadvantaged students, to high quality schools ( Zancajo, 2019). However, empirical evidence highlights the opposite. Socioeconomic status influences the ability and willingness to exploit school choice, while private school selection practices maintain segregation (2019: 3).
- Verger *et al*.’s (2020) scoping review of public private partnerships (PPP) suggests that Chile’s results are generalisable: ‘PPPs generate a trade-off among social equity and academic achievement. Thus, if the aim of educational policy is to promote inclusion and equity, the implementation of most of the PPP programmes analysed in this paper would not be advisable’ (2020: 298).

### 2. Competing definitions and alternative researcher aspirations

Eng (2016: 676–7) highlights the major disconnect between: (1) research showing that social determinants are far more important to attainment than school performance; and, (2) the US public and policymaker tendency to see this issue in terms of individual merit and school or teacher performance. Eng’s (2016: 683–6) solution is more effective framing, to emphasise the benefits of a ‘systems approach’ and ‘collective action’ to counter ‘individualistic thinking’.

More generally, research recommendations include to: avoid narrow definitions of equity associated with school performance and testing; foster more inclusive and deliberative dialogue between school leaders, teachers, and communities to co-produce meanings of equity; recognise how multiple forms of inequality and marginalisation reinforce each other; ‘treat race as an urgent marginalising factor’ and gather specific data to measure racialised outcomes ( George *et al*., 2020) rather than hiding behind ‘colour blind’ or ‘race neutral’ strategies ( Felix & Trinidad, 2020; Li, 2019; McDermott *et al*., 2014); and, provide proper resources to address sex discrimination ( Meyer *et al*., 2018).

In that context, Thorius & Maxcy (2015: 118) describe ‘six transformational goals’ for ‘equity-minded policy’: ‘(a) equitable development and distribution of resources, (b) shared governance and decision making, (c) robust infrastructure (e.g., efficient use of space and time), (d) strong relationships with families and community members, (e) cultures of continuous improvement, and (f) explicit emphases on equity’. Multiple studies use such goals to set standards for policy reforms.

***Finland’s comprehensive model.*** Finland has an international reputation for pursuing equity via lifelong learning and a comprehensive schooling system, supported by a Nordic welfare state ( Grek, 2009: 28; 33; Lingard, 2010: 139–40; Niemi & Isopahkala-Bouret, 2015). Equity means ‘minimising the influence of social class, gender, or ethnicity on educational outcomes’ while making sure that everyone achieves a *threshold* of basic education and skills via:

1. ‘active social investment through universal early childhood education’
2. ‘a comprehensive education model’ in which every school has a near-identical standard
3. ‘the provision of support to lower-performing or at-risk students’
4. resistance to neoliberal, market-based reforms that foster individualism and competition ( Chong, 2018: 502–5).

Consequently, it has enjoyed high praise from the OECD for minimising the number of people leaving school without adequate skills ( Field *et al*., 2007: 26) *and* from researchers who welcome a focus on social determinants (although the focus on an equity threshold contrasts with approaches to health - Cairney *et al*., 2021).

***Equity-oriented leadership: education debts and recognition.*** Farley *et al*. (2019: 2) examine how ‘leadership standards … represent evolving conceptions of equity and justice’. The context is a drive in the US for schools led by ‘equity-oriented change agents’ (2019: 4). It is undermined by a tendency for equity and justice to be defined in different ways in professional standards and training, with too few connections to social justice research and too many individualist conceptions of achievement gaps (2019: 9). Problems of inequity are ‘wicked’ and ‘relentless’, and not amenable to simplistic solutions based on ‘equal access to resources, curriculum, or opportunities’ (2019: 15). Rather, Farley *et al*. (2019: 9) laud Ladson-Billings’ (2008); Ladson-Billings’ (2013) idea of an ‘education debt’ in which all members of society should consider their contribution to inequalities and challenge the sense that the ‘attainment gap’ is inevitable (see also Horn, 2018: 387). They seek *recognition* approaches that ‘characterize injustice as both structural and as an inherent failure of society to recognize and respect social groups’, including ‘the way that individual actors can oppress groups via exploitation, marginalization, powerlessness, cultural imperialism, and violence’ ( Farley *et al*., 2019: 10):

‘These educators foreground their commitment to social justice and equity and avoid deficit views - and they also reflect those values in their practice. They take part in courageous and vulnerable conversations, persist in working to remove inequities, and respect and appreciate the assets within their students and their communities’ (2019: 3).

Similarly, Feldman & Winchester (2015: 69–71) distinguish between (a) the limited-impact formal measures that establish legal rights, and (b) policy designs grounded in practice and continuous discussion - ‘courageous conversations’- over many years. Crucially, policy does not have a settled definition of equity. It is to be negotiated in practice as part of an inclusive approach to policymaking, backed by the commitment of school districts to ‘owning past inequity, including highlighting inequities in system and culture’ and ‘foregrounding equity, including increasing availability and transparency of data’ ( Rorrer *et al*., 2008: 328).

***Challenging the ‘colour evasiveness’ of ‘equity for all’ initiatives.*** Felix & Fernandez Castro (2018: 1) examine the Student Equity Plans that Californian community colleges are obliged to produce, to identify how they operationalise equity. This focus is significant since there is highly unequal access to elite universities in favour of white populations ( Baker, 2019), and public and private research universities spend double per student than community colleges ( Felix & Fernandez Castro, 2018: 3). The latter ‘enrol a larger proportion of low-income, first-generation, and racially minoritized students [70% of students of colour]… a disproportionate number of students who have faced constant disadvantage and inequality throughout their educational trajectory’ (2018: 3), and their dropout rates are far higher (2018: 3–4).

In that context, are colleges race-conscious, and do they hold practitioners and institutions - rather than students - responsible for the pursuit of equitable outcomes? They find that few (28/178) plans ‘explicitly targeted Black and Latinx students with culturally relevant, data-driven, evidence-based strategies’, partly because funding incentives for equity plans only appeared in 2014 (2018: 2) and because California rejected (via general election ballot) ‘affirmative action’ policies ( Baker, 2019; Felix & Trinidad, 2020: 466). Instead, there is a tendency to produce ‘equity for all’ messages to address disadvantaged groups – related to ‘race/ethnicity, gender, veteran status, foster youth, socioeconomic status, and ability status’ - rather than addressing racism or social determinants of education ( Felix & Fernandez Castro, 2018: 7–9; 24). This outcome relates strongly to ‘interest convergence’: when white people only agree to policies benefiting racialised minorities if they too benefit ( Felix & Trinidad, 2020: 470). Or, schemes have a faulty logic, such as the ill-fated financial incentive to complete 100 hours of community work ($4,000 towards college tuition) which supports the white and relatively affluent students who can afford to work without pay ( Wells & Lynch, 2014)

Similarly, McDermott *et al*. (2014: 541) use US case studies of ‘student assignment policy’ to show that ‘race-neutral policies seem to generate the opposite dynamic’. The context is of historic problems with desegregation policies designed to address the highly unequal quality of schools available to white and black students. They prompted a trend towards ‘race-neutral politics’, focusing on addressing socio-economic issues rather than race, to make policy changes less vulnerable to legal and political challenge by the white majority. Policy helps to reduce overt bigotry but also hide and exacerbate racialised disparities because: (a) a focus on less-advantaged and needier students allows white parents to oppose their inclusion without referring to race, (b) people can oppose ‘busing’ children to school with reference to cost, and (c) people seeking ‘enclave’ schools can refer to the common sense of neighbourhood schools rather than keeping out black children (2015: 541–3).

***Fostering a ‘capabilities’ approach.*** Multiple studies highlight measures taken in the name of equity which exacerbate or fail to reduce inequalities. In New Zealand, removing tertiary education fees without addressing inequalities of debt or ability to attend, while providing superficial support to tailor schooling to Maori culture, produces the veneer of equity but unequal outcomes ( Barker & Wood, 2019). In many sub-Saharan African states, unequal access to high quality HE is exacerbated by multiple and intersecting sources of disadvantage and marginalisation, despite the pursuit of equity initiatives by UNESCO, the World Bank, the African Union, the African Development Bank, and the Association of African Universities ( Singh, 2011). 

In that context, some studies draw on Sen (1999); Sen (2009) and Nussbaum (2000) to highlight a ‘capabilities approach’. While neoliberal approaches require individuals to adapt to school requirements, a capabilities approach fosters a learning environment more tailored to their needs and more able to empower them to learn ( Wahlström, 2014). It incorporates the ability of people to take up opportunities to learn when they are subject to differences in power, culture, and resources.

Molla & Gale (2015: 383) apply this approach to HE ‘revitalization’ in Ethopia, driven by ‘social equity goals’ and ‘knowledge-driven poverty reduction’ (encouraged by the World Bank). They found that equity policies included a commitment to address previous ethnic injustices, targets and resources to enable disadvantaged groups to enrol, lower entry requirements for disadvantaged groups, and expansion from two to 32 universities and from 20k to 250k students by 2012 (using the private sector to fund expansion) (2015: 385–6). Yet, ‘the problem of inequality has persisted along the lines of ethnicity, gender, rurality and socio-economic background’ (2015: 383). For example, women represent 26.6% of enrolled undergraduate (20% postgraduate taught, 17% PhD), concentrated in non-STEM subjects, and with higher attrition rates linked strongly to sexual harassment and assault by male teachers and students (2015: 388; compare with Wadesango *et al*., 2011 on schools in South Africa). There are also geographical variations in school completion/HE eligibility, and ‘over 70% of students in Ethiopian HE come from families in the top income quartile and from urban areas’ (2015: 387).

Molla & Gale (2015: 383) identify the lack of attention to ‘a deprivation of opportunities and freedom’. A focus on capabilities emphasises the role of education in wellbeing and freedom: the ability to read, write, think, and deliberate contributes to self and external respect and access to further educational and economic opportunities. It highlights the barriers and opportunities to that freedom, including ‘structural constraints (embedded in policies, curricula, pedagogical arrangements, social relations and institutional practices) that limit agency freedom and deny social groups recognition and respect’ ( Molla & Gale, 2015: 389–90). Progress requires agency to ‘convert’ resources and opportunities into processes and outcomes: ‘repressive cultural values of society and public policy inactions influence people’s subjective preferences and constrain their real opportunities to choose, and thereby create and sustain inequality’ (2015: 390). This is about the fairness of allocation *and* the *relevance* of opportunities to each person or group, subject to their levels of repression, poverty, and geography.

***Policymaking contradictions: neoliberalism versus social justice.*** Hajisoteriou & Angelides (2020) describe competing definitions of education equity as neoliberal versus social justice, which interact to produce often-contradictory approaches. They describe global policymaking as two-headed: ‘beyond the rise of hyber-liberalism, xenophobia and socio-economic inequity, globalisation has also humanistic and democratic elements” (2020: 282). Globalisation has helped produce ‘global policies of social justice and equity’ as well as increased migration, and ‘may play a substantial role in the development of minority and immigrant rights, while also moving citizenship debates beyond the idea of the nation state’ (2020: 278; 282). There is also a dominant discourse on human capital and global economic competitiveness, combined with NPM techniques:

‘international benchmarking, the privatisation of education, importing management techniques from the corporate sector and other ideals such as choice, competition and decentralisation … school-based management, teachers’ accountability, public-private partnerships and conditional fund-transfer schemes are some of the global education policies often cited as a result of neoliberalism’ (2020: 277).

This dominance has a profound impact on professional practice, at the expense of social justice:

“global discourses of social justice and equity of educational opportunity appear to be often counteracted by global discourses of neoliberalism, which are embedded in international performance indicators, and international tests and scores. Market oriented education seems to overrule policy reforms aiming to achieve equity in education …[producing] educational policies preoccupied with efficiency, ‘excellence’, ‘standards’ and ‘accountability’" (2020: 282; 277).

It extends to the classroom, pressuring teachers “to become classroom ‘technicians’ whose quality is defined in terms of testable content knowledge instead of professional knowledge”, limiting their ability to promote a social justice approach to education as ‘critical thinkers, active professionals and thus agents of change’ (2020: 283; Klees & Qargha, 2014: 323).

***Variable country and regional experiences of neoliberal policies.*** Many country and regional studies make the similar argument that ‘central neoliberal technologies of accountability, competition, privatization, marketization, managerialism, and performativity’ undermine equity initiatives ( Clarke, 2012: 176). However, the effect is not uniform ( Novak & Carlbaum, 2017: 673). There is a spectrum of cases in which neoliberal ideas are dominant or resisted.

For example, neoliberalism is the established order in *the US*, and studies suggest that a market-driven narrative undermines a social determinants focus ( Chu, 2019). Further, studies of *Australia and New Zealand* present a similar assumption that neoliberal approaches have long dominated education policies. Clarke (2012: 172) describes a tendency for Australian (and many other) governments to embrace neoliberal approaches to globalisation, emphasising individualism and markets, and situating education policy and the measurement of a country’s educational performance in that context (2012: 175). A focus on education for the economy dominates, with social justice programmes treated as bolt-ons and band-aids (2012: 176; see also Angus *et al*., 2013: 563; Loughland & Sriprakash, 2016: 230; Morsy *et al*., 2014; Taylor, 2004: 439–40). Worryingly low trust in, and respect for, teachers reflects New Zealand’s contradictory ‘neoliberal education policy which has pushed for simultaneous devolution and control, marketisation and competition for more than 30 years’ ( Barker & Wood, 2019: 239).

Canadian experiences are *somewhat* different, since Mindzak (2015: 112) relates the lack of US-style charter schools (run by private boards) to a relatively communitarian ‘commitment to equity’ built on ‘an overarching belief in the moral rightness of public systems of education in Canada’, a tendency for more equitable funding for schools (across and within provinces), and a wider commitment to the welfare state. Further, ‘Toronto has rejected many exported reforms from the United States, such as high-stakes standardized examinations, school sanctions for low performance, value-added evaluations of teachers, and charter school and voucher programmes’ ( Hamlin & Davies, 2016: 190). Regional and country studies describe the threat of neoliberalism to a more communitarian history, and the inherent contradictions in Canadian policy rhetoric. Most identify the alleged-but-unfulfilled expectation that market-led initiatives (vouchers and school choice) would reduce education inequalities, some highlight their contribution to the neglect of anti-racist policies, and some describe multiculturalism as a tool of economic policy (e.g., Fallon & Paquette, 2009; Gulson & Webb, 2013; Martino & Rezai-Rashti, 2013; Paquette, 2001; Segeren & Kutsyuruba, 2012; Winton, 2018).

Similarly, Nordic discussions describe the threat of neoliberalism to social democratic values built on trust and social capital (and comprehensive non-selective education), but with Scandinavian countries further down the road than Finland ( Chong, 2018: 502). Engsig & Johnstone (2015: 472) argue that the focus on student higher-stakes testing to aid performance management-driven quality improvement (coupled with a reduction in funding per student) was ‘directly inspired’ by US policy (2015: 472).

Varjo *et al*. (2018: 481; 483) compare how Finnish and Swedish local education authorities deal with major changes to the Nordic model, combining decentralization, market-based reforms, and some evidence of greater segregation ‘along ethnic and socio-economic lines’ following the introduction of school choice policies. In Finland, decentralization is in the context of the maintenance of comprehensive schooling and no tradition of ‘mandatory national testing … school inspections and school league tables’ (2018: 486). School assessments remain unpublished to prevent media stories of the ‘weakest’ schools (2018: 489).

In contrast, Swedish governments encouraged a larger private sector: 26% of students in 2015 attended government-subsidised private schools, with a marked spread by geography (50% in large cities, 3% in rural areas) and class (55% in highest and 5% in lowest socio-economic decile). They fostered school choice via vouchers for students (although elite schools have long queues) (2018: 486–8). It contributed to a data-led competition between state and private schools (2018: 489). There is also evidence of rural student commutes to cities but not the other way, prompting some rural schools to sell themselves as more welcoming to local immigrant populations (2018: 490–1). The reforms also produced tensions between the *trust in* versus *audit of* teachers in Sweden when checking how fairly they grade national student tests ( Novak & Carlbaum, 2017: 673). The choice to introduce an Inspectorate and regrading programme contributed to a government and media narrative on ‘teachers’ assessments as incorrect, unfair and as jeopardizing the credibility of the grading system, thus justifying increased central control and authority over teacher assessments’ (2017: 673; Wahlström, 2014).

Camphuijsen *et al*. (2020: 4) identify comparable developments of ‘test-based accountability (TBA)’ in Norway, which previously seemed ‘immune’ to neoliberal agendas since it maintained a social democratic welfare state and comprehensive education system with strict limits on private schools and school choice. Indeed, while an OECD report in 1988 questioned its ability to hold a decentralised school system to account, reforms were largely resisted by ‘key political actors, parliamentarians and the main teacher’s union’ (2020: 5). Things changed following the ‘PISA shock’: poor performances in PISA 2000 and 2003 ruined Norway’s self-image as ‘the best school in the world’, highlighted inequitable outcomes, and showed that 17% of students left school without basic competencies (2020: 7). The reform-push coincided with rising NPM and outcome-based management (encouraged by the OECD). Further, TBA’s longevity was assured when it became all things to all people: an equity measure for some, and for others ‘a means of scapegoating teachers, school leaders and local authorities’ (2020: 12).

In each country, while state spending per capita on education may be crucial, few studies provide detailed and systematic accounts of the role of unequal spending across regions. One exception is Garritzmann *et al.* (2021: 3) who produce new ‘data on regional per capita public education spending in 282 regions in 15 OECD countries over two decades (1990–2010)’ to identify a wide range of unequal regional spending. They find that left-wing governments are more likely to increase education spending, not only at a national level but also in regions with significant powers. As such, the countries most conducive to regional government impacts are Canada, the US, Germany, and the UK, followed by all Swiss, most Belgian, and few Italian regions (2021: 20).

There are generally fewer studies of Global South experiences. Most accounts highlight the impact of unequal global power relationships, in which a small number of international organisations and Global North countries promote neoliberal global agendas with a major impact on policy in Global South countries. For example, Spreen & Vally (2010: 429) contrast domestic South African equity initiatives versus the international neoliberal agendas that focus more on economic frames (2010: 429). The initial context was a post-Apartheid period built on hope that a new system would encourage more equity via a focus on rights, boosted by an idealised notion of education and teachers, without considering what it takes to transform policy and outcomes, the implementation challenges, and the path dependence of the old system. When attention shifted to fundamental reforms, policymakers oversaw ‘a careful balancing act between contradictory political imperatives, chiefly social justice and economic development’ (2010: 435–7). There was ‘growing criticism and pressure to increase quality, improve access, equity and accountability’ (2010: 431), prompting policymakers to rely on economic and management experts, not the knowledge of local communities and the vulnerable populations most deserving of government support (2010: 445). While much explanation comes from global economic pressure, and international organisation agendas, this approach was also a choice by domestic policymakers to connect education to economic growth rather than to address poverty. Like ‘most western countries’, periods of economic crisis prompted a focus on austerity and economic growth (2010: 429–30).

## Policymaking complexity: top-down and bottom-up approaches

Policy studies highlight a strong connection between policy ambiguity and policymaking processes, with the latter commonly described in relation to complex systems or environments that are out of the control of policymakers ( Cairney, 2020). While governments or international organisations may decide how to define equity, they do not have the power to simply turn their definitions into policy outcomes. Outcomes seem to ‘emerge’ from local interactions, often in the absence of central control. Further, since policy is so interconnected, the impact of one agenda can amplify or undermine another ( Cairney *et al*., 2020).

In that context, a recurring theme in our review is the tension between two often-contradictory aims:

1. To *centralise*. To prioritise a common purpose, directed from a single authority and formalised in multiple levels of government, expecting fidelity to a general aim of reducing unfair inequalities.
2. To *decentralise*. To prioritise the legitimacy of multiple forms of governance, directed by local policy actors in collaboration with stakeholders and communities to make sense of policy aims, expecting that the results will be different from a central agenda.

This tension is apparent in the previous section: centralised approaches to setting standards, performance management, and accountability exist in tension with decentralised approaches to local government and professional autonomy. If policymaking is centralised *and* decentralised, we cannot understand one without making sense of the other. 

The classic way to describe such dynamics is ‘top-down versus bottom-up’ approaches to implementation studies ( Cairney, 2020: 30). In HiAP studies, researchers tend to apply a top-down lens to describe ‘implementation gaps’ ( Cairney *et al*., 2021). In education research, local sense-making among ‘street level’ ( Lipsky, 1980) practitioners matters. Studies provide insights on policymaking by treating participants as legitimate policy actors rather than obstacles to delivering a top-down agenda. Nevertheless, there is some debate on the extent to which central or local direction is more conducive to equity.

### Top-down explanations for limited progress on equity (the US)

Chu (2019: 3) describes initiatives over five decades to address ‘equity, or inequity, on the basis of race, ethnicity, socioeconomic status, language, able-ness, gender, sexual orientation, and immigration status’. Yet, there remain ‘persisting and exacerbating disparities in educational opportunity and outcome between more privileged students and students from marginalized and minoritized groups’. Chu (2019) relates this gap to vague ambitions and contradictory policies. State governments define equity vaguely in their Every Student Succeeds Act 2015 (ESSA) strategy documents, and few plans describe how to achieve it (2019: 3). In *practice*, most relate equity to ‘equitable access to educational resources - including funding and effective educators’, under half ‘attend to equity in outcomes’, most relate outcomes to a threshold of performance (measured by ‘student standardized test performance’, and this ‘adequacy-based view of equity has been favored by court rulings and embraced by many policy makers and district and school leaders’ (2019: 5).

This lack of clarity minimises attention to a faulty premise for policy design: the assumption that equity in outcomes results from a commitment to funding and teachers. The ‘teachers matter’ mantra draws attention from racism and a tendency for poor-income areas to provide less funding for schools via local taxes. It exacerbates other problems, such as when ‘falling behind’ schools have to focus more on teaching-to-the-test to show progress (2019: 20). It favours neoliberalism and undermines a social determinants focus:

‘by regulating that every student should be equitably taught by experienced and effective teachers who are certified to teach in the subject areas, the concept of equity is also implicitly tied to the values of productivity, cost-effectiveness, human resources management, and economic return of investment that are essential to the neoliberal, market economy … The democratic and social significance of education is thus given less attention’ (2019: 21).

Multiple studies also argue that a focus on teachers and performance pretends to be meritocratic and equitable, but undermines attention to unequally distributed resources ( Bishop & Noguera, 2019: 124; Evans, 2009) and exacerbates inequalities: ‘Whether viewed from a perspective of unequal resources, testing bias, or technical flaws, the proficiency game is rigged’ ( Horn, 2018: 387). There are tensions between ‘compliance’ with strict centralized accountability measures versus the ‘innovation’ needed in ‘alternative schools’ to produce more deliberative equity strategies ( Hemmer *et al.,* 2013: 655–6; Trujillo, 2012; see also Jimerson & Childs, 2015). Further, school and district leaders know how to play the game of talking up social justice while everyone knows that their performance will be measured according to school performance in ‘achievement gap initiatives’:

‘Aspiring administrators are learning this logic and are taking and passing the tests. It is as if they know the overarching policy logic is to compete and measure up, but they learn the talk of equity, community, diversity, and inclusion’ ( Marshall & McCarthy, 2002: 498).

Lenhoff (2020) describes similar problems with the illusion of greater equity in relation to access to a preferred school. School choice policies *appear* to reduce segregation but really introduce new ways to compete unequally. In theory, choice produces a competitive market, with schools having to offer better quality to compete (2020: 248–9). Further, ‘decreasing the number of racially and economically segregated schools and increasing access to schools with lower rates of poverty and more racial diversity are essential to ensuring that public education serves all students equitably’ (2020: 252). In practice, black students are more likely to attend local ‘low quality’ schools since their parents have fewer resources to fund travel and navigate the complex admissions procedures (often designed to reduce demand) and lower confidence that their child would be accepted. Further, residents’ influence over selection for ‘high quality’ schools help maintain a predominantly white population. While an incentive to accept students according to funding formulas may help, it also prompts schools to find new ways to restrict access (2020: 250).

### Bottom-up and ‘sense making’ explanations for limited progress (the US)

US studies – primarily of race, minoritization, and socio-economic inequalities - examine the extent to which local policy actors constrain or facilitate equity policies when making sense of central and local initiatives (citing Coburn, 2001; Coburn, 2005; Spillane *et al.*, 2002). Several draw on Oakes *et al.* (2005) to describe a ‘zone of mediation’ that influences proposed policy changes.

Oakes *et al*. (2005: 283) examined attempts to detrack and ‘find more equitable ways to distribute resources and educational opportunities’ in ‘a racially and economically diverse school setting’. District and school leaders ‘saw themselves as change agents spearheading an ongoing process of improvement’, encouraging changes to norms via open and regular conversations on progress. However, they recognised that no policy change happens as planned, since it involves the interaction between new ideas and established cultures and practices (2005: 284). Most leaders described negative experiences of attempts to ‘give more to our least powerful citizens - low-income and non-white students - in a societal culture that usually demands that they receive less’. Parents of white children often complained – successfully – that more-inclusive reforms, to give some students longer to complete modules or expand access to advanced modules, would reduce their children’s ‘high status associated with more exclusive classes’ (2005: 286). The experience is short and dispiriting if leaders are unprepared for the backlash:

‘the majority of change agents in our schools had little reason to suspect that deeply held beliefs and ideologies about intelligence, racial differences, social stratification, white supremacy, and elite privilege would penetrate their local discussions of educational reform. … Most naively proceeded as if support for equity reforms would emerge if only they could provide “evidence” that detracking enhanced the achievement of struggling students without harming their traditionally successful peers’ (2005: 287).

Policy change in each school or community relates to a ‘zone of mediation’ that includes levels of ‘tolerance’ for change and the ‘larger cultural norms, rules, values, and power relations’ which ‘promote either stability or change’ (2005: 288). Influences range from ‘global capitalism’ to campaigns to defend the current distribution of resources. The latter emerge from parental or staff beliefs that non-white children are less intelligent or that poor parenting undermines achievement (2005: 294). In that context, top-down mandates may be necessary but insufficient to ensure major policy change (2005: 297; compare with Michener, 2019).

Some articles provide a more optimistic (or mixed) assessment of the impact of leadership. For example, Halverson & Plecki (2015) describe a window of opportunity for a school district to overcome ‘reform fatigue’ and manage parental opposition while trying to reframe and prioritise equity. School leaders used the prospect of major demographic shifts – a widening socio-economic gap between schools, linked to increasing segregation in local communities – to foster vertical equity in resource allocation. Still, it took *almost a decade* of district-led participation with teachers and parents ‘to define, articulate, implement, and sustain their commitment to closing the achievement gap and improving learning for all students’, which included generating a more positive social construction of minoritized students and avoiding the sense among non-targeted schools that benefits are only available to targeted schools (2015: 55; see also Wang, 2018: 409 on the subversive tactics of school principals in Canada).

Feldman & Winchester (2015: 66–7) describe the potential for leadership to make a difference if education reformers ‘refocus policy implementation research’ to take local practices and mediation seriously, while Rorrer *et al.* (2008: 328) argue that school ‘districts are also capable of disrupting and even displacing … institutionalized structures and practices that perpetuate inequity in student achievement’. Brezicha & Hopkins (2016) use the ‘zone of mediation’ to highlight the positive impact of a community organisation that offered translation services and English-language teaching for Dominican parents.

Rosen & Mehan (2003: 678–80) show that the architects of a new Charter School (to connect the University of California, San Diego to its local community) navigated successfully the constraints of a ban on affirmative action. Its focus on helping low income students, working hard to gain entry to elite HE, helped generate support from right-wing politicians focusing on individual motivation and left-wing politicians who appreciated a work-around that focused primarily on students of colour. In comparison, Neumerski & Cohen (2019) find that fee-paying schools (Catholic, Montessori, and International Baccalaureate) have found it difficult to maintain their traditional focus on ‘excellence’ (to maintain market share) with ‘equity’ (to teach poorer students in less advantaged areas) when responding to US policy reforms and demographic change. Finally, Superfine & Thompson (2016) describe the positive potential of US courts, in this case to protect teacher tenure rules to avoid the ‘disproportionate provision of underqualified and ineffective teachers to minority and/or low-income students’ (2016: 573–4).

However, most US articles suggest that advocates for change are swimming against the tide. Thorius & Maxcy (2015: 119) use the ‘zone of mediation’ to analyse ‘tensions between new and old ways of doing things’ when ‘federal special and general education policies intersect in local sites’. They examine the 1997 revisions to the US Individuals with Disabilities Education Act (IDEA), designed to reduce the inappropriate use of ‘special education eligibility and placement’. It took forward the ‘Least Restrictive Environment (LRE) mandate’ on mainstreaming education that emerged from the civil rights movement and court actions to secure access to public education (2015: 116). However, it emerged in a ‘policy landscape that has moved rapidly toward emphases on efficiency, standardization of learning outcomes, and accountability measures that prioritize test scores over student development’ (2015: 116). This potential for two initiatives to collide took place in the context of high professional discretion to interpret criteria to determine who has learning disabilities. The result was historically high categorisations of disability in relation to ‘students of colour’ and ‘English language learners’, exacerbating a tendency for minoritized students to be ‘disproportionately placed within more restrictive educational placements’ (2015: 117; Schuelka, 2013). Welsh & Little (2018: 753) find comparable patterns in school discipline measures: ‘Exclusionary discipline policies and practices disproportionately affect African American students and leave these students most vulnerable for entry into the school-to-prison pipeline’.

Multiple US studies highlight similar outcomes when school leaders and teachers make sense of contradictory equity initiatives. Turner & Spain (2020: 786) examine the potential for US school districts to (a) overcome the parental opposition to detracking reforms described by Oakes *et al.* (2005), and (b) make such action consistent with wider agendas, such as to close ‘achievement gaps’. Administrators criticised tracking as ‘contrary to a democratic ideal of equal access to educational opportunities’ and ‘a constraint on their efforts to address state and federal educational policy goals’ (2020: 794). However, they also used the language of ‘gifted students and the achievement gap, individualization, and excellence for all’ to connect their aims to a dominant discourse. Such ‘colour-blind’ terms normalise white middle-class equity frames by obscuring ‘the historical, systemic roots of underachievement’ in relation to ‘systemic school and social inequalities’, but leaders find them useful to make a case for change (2020: 794). Still, they could not find a discursive strategy to overcome opposition and ‘they largely left tracking structures in place’ (2020: 804).

Turner’s (2015) case study of district leader sense-making identifies their tendency to relate demographic shifts (rising poverty and immigration, and ‘increasing populations of students of colour’) to their worries about ‘white flight’ if their social justice policies are too energetic. Turner (2015: 29) finds a mixture of positive intentions (including to address out of school factors) *and* negative stereotypes regarding the deficits of students in relation to English-language speaking or parental support. The result is a tendency to argue that other people are racist, while avoiding talking about the structural causes of racial disparities or finding ways to empower or celebrate the value of students of colour.

Horn (2018) and Bishop & Noguera (2019) examine the unequal impact of ESSA’s predecessor: the No Child Left Behind Act 2001 (NCLB). NCLB received bipartisan congressional support to reduce an ‘achievement gap’ associated with ‘racial and socioeconomic disparities in academic performance’ within schools, but there remain ‘large and persistent disparities’ (2019: 122). Bishop & Noguera (2019: 123–6) relate failure to insufficient attention to the ‘pervasive structural inequities in schools and societal factors outside of schools’, or the ‘out of school’ factors such as parental income and wealth, health and healthcare, nutrition, and physical and social environment (see also Pelletier & Manna, 2017). Instead, NCLB initiatives rely on schools to close the gap, while overseeing major inequalities of funding, and high stakes testing to manage performance.

Similarly, Horn (2018: 383) highlights a dichotomy between schools. Some are wealthy and with children from wealthy backgrounds, which largely insulates them from NCLB consequences. Others are low resourced, in poor areas, teaching marginalised students, and disproportionately vulnerable to the ‘consequences for not performing satisfactorily … a loss of autonomy via reconstitution, chartering, or state takeover’ (Horn, 2018: 383). In that context, Horn (2018: 401–3) uses case studies of school practice to identify the inequitable consequences of street-level behaviour: some students are labelled as poor competence, attitude, and behaviour, and subjected to testing to meet basic requirements; others are labelled as worthy of investments for useful learning. Thus, ironically or intentionally, a ‘colour-blind, techno-rational policy discourse’ helps to exacerbate ‘the very inequality that NCLB seeks to remedy ... policy often intensifies the problems it purports to address’ (2018: 384).

Blaise (2018: 1154) also highlights contradictions in equity initiatives focusing on high stakes testing. In this case study, of a high school graduation exam, Haitian students get no extra time to adjust to a new education system or learn English to the required standard, so perform badly in relation to a policy designed ostensibly to foster equitable outcomes.

Additional studies present variations on this theme of tracking based on a deficit model of students and their parents. Park *et al.* (2012: 669) suggest that school leaders (in a Californian district) make sense of performance data through the lens of a ‘deficit model’ of ‘low students’ (2012: 669). Porras (2019: 227–9) shows how they exacerbate this problem by failing to involve ‘Latina immigrant mothers (mamás)’ in parental and community forums. Bertrand *et al.* (2018) describe a tendency among (generally white) school principals to view ‘parents of color and working class parents … in terms of deficiencies and as needing to learn to better support school goals’ (2018: 1). As such, they do not harness the potential for democratic deliberation when engaging to discuss school improvement processes (2018: 5). Hara (2017: 466) relates limited policy change to teachers who ensure that practices are ‘significantly different from the written policy developed at federal, state, and local levels’ (in this case, teacher trainees had minimal knowledge of second language issues). Overall, there is limited thinking about the social determinants of education, exacerbated by low teacher knowledge of equity policies.

Such outcomes are reinforced by unequal financing and opposition to policy change. Donaldson *et al.* (2016: 185) describe the unequal impact of US initiatives to improve equal access to high quality teaching (e.g., the federal ‘Race to the Top initiative’). Higher resourced schools, with less need to address poverty, have more access to good information on teaching evaluation and tend to benefit more from the reforms, while ‘teachers at schools enrolling greater numbers of low-income students and students of color received less robust opportunities to learn’ (2016: 198). Further, advocates of the non-governmental ‘Common Core’ initiative (designed so that ‘higher, common standards will yield universal college-and-career readiness’) describe intense opposition by ‘parents, members of local communities and school boards, and educators’ who saw it as back-door for federal government ‘3 ^rd^ wave’ reforms based on ‘performance management via testing for educational outcomes’ ( Kornhaber *et al.,* 2017: 404). Eng (2016: 681) argues that this outcome resulted from its poor framing by advocates.

### Wider international implementation experiences

The US seems to be a relatively extreme case in which individualisation, backed by market forces, trumps state intervention to address structural issues. Still, other country studies have a similar focus on implementation gaps through a top-down or bottom-up lens. Global South studies also highlight the exacerbating impact of external influence.

### The failure of externally driven neoliberal ‘whole system’ reforms

De Lisle (2012: 68) draws on ‘postcolonial and small state theories’ to analyse limited progress towards ‘whole system reform’ to improve access to high quality secondary education in Trinidad and Tobago. The context is of high external influence on policy, caused by the (1) legacy of colonialism (the maintenance of UK grammar schools) and (2) tendency for reforms to be funded and directed by international organisations (e.g. the Inter-American Development Bank, IDB) rather than the domestic government (2012: 68). Implementation analysis helps identify common problems, including; reform ambiguity; insufficient collaboration, communication, stakeholder involvement; and too-limited leadership and planning (2012: 71–6). Further, De Lisle (2012) reminds influential donors that a top-down approach and lack of attention to country and local context exacerbates policy failure.

Singh (2011: 355–7) relates a major implementation gap – on ‘access, equity and quality in African higher education’ – to a tendency to model sub-Saharan African HE in relation to colonial country provision, backed by World Bank quality assurance measures.

Spreen & Vally (2010: 445) relate the slow progress of South African school equity reforms to the excessive focus on global neoliberal policy agendas at the expense of incorporating ‘the needs, understandings and social realities of its primary constituencies’ (see also Wadesango *et al*., 2011, comparing equal access but unequal treatment for girls within schools). Further, Wiseman & Davidson (2021: 1) argue that reformers ‘cloak’ policy change in equity language, and use quantification to depoliticise reforms, while placing the onus on individuals rather than the state (2021: 3; 12). Meanwhile, ‘neoliberal education policies’ are ‘positioned as post-apartheid equalizers’, built on three profoundly misleading claims that: (1) ‘school choice’ would help end historic racial segregation; (2) school fees would help redistribute funding to poorer schools (rather than limit access to the most-resourced schools); and (3) high-stakes national testing would help ‘develop a more equitable education’ (2021: 7–10).

In mild contrast, Yazan (2014) describes the role of international organisations – including the EU and UNICEF – as essential to increase the number of girls attending schools in Turkey. In particular, UNICEF funding made projects seem financially feasible enough to ‘survive in the policymaking process’ (2014: 847).

### The lack of an implementation strategy (Tanzania, El Salvador)

Mtahabwa (2010: 353) contrasts (1) a government’s formalised commitment to ‘preschool education as a basic human right in Tanzania’, with (2) lack of an ‘implementation plan to facilitate translation of the policy into practice’ (2010: 361). Similarly, Edwards Jr *et al.* (2015) explain the limited implementation of gender equality policy in El Salvador (school access for girls) by contrasting high social movement and civil society support for policy change (aided by international organisation funding) with low Ministry of Education attention to an implementation strategy.

### Federal multicultural policies mediated by Canadian provinces

Segeren & Kutsyuruba (2012: 1) relate the ‘oft-cited inadequacies of the policies and pedagogies of multicultural education’ in Ontario to limited implementation. Federal government policies were subject to ‘slow and uneven implementation, cautious adaptation, inaction, and even outright rejection’ (2012: 2). This mediating role contributed to ‘the development of few policies in the area of equity and inclusion, whereas developed policies have had only minor impacts’ (2012: 2).

### Top-down multicultural policies diluted during implementation (Cyprus)

Hajisoteriou & Angelides (2014: 157) contrast the discourse of government documents with the practices of schools and teachers. Vagueness in government aims (to respect ‘diversity and cultural, linguistic and religious pluralism’) ensures that schools reproduce ‘cultural domination, non-recognition and disrespect’ and do not adapt their equity policies to the social background or cultural practices of marginalised students: ‘policy-makers themselves do not value their own policy rhetoric for social justice, thus failing to get schools to take such policy priorities seriously’ (2014: 159; 168).

### Bottom-up policymaking in a top-down context (England, Wales, Scotland)

Chapman & Ainscow (2019: 899) relate the ‘equity policy challenge’ to case studies of ‘bottom-up leadership within a context of top-down political mandate’. They highlight (1) the routine use of centralised accountability measures regarding quality and performance, and central government drives to improve school management and place high quality teachers in schools in ‘disadvantaged communities’ (backed in Scotland by a ‘Pupil Equity Fund’), and (2) national-local government tensions in relation to who should drive the agenda and how much variation in processes to tolerate (2019: 899; 909).

### The strategies of school leaders (Australia)

‘In the Adelaide Declaration, the Council of Australian Governments … set out to address the effects of socio-economic status, geographical location, Indigeneity, and other social categories on educational opportunities and learning outcomes of students’ ( Molla & Gale, 2019: 858). Yet, disparities persist. Molla & Gale (2019: 858) relate this gap to school leaders using their discretion while implementing national government equity policies. Their school’s resources and ‘institutional ethos’, and their own ‘social justice dispositions’ influence their stances (2019: 858). Responses range from: *compliance*, or implementing policy when your job may be on the line (in ‘disadvantaged public schools’ dependent on state funding), to *compromise*, or mediating policy when subject to encouragement rather than imposition (private schools selecting who gets means-tested scholarships), and *contest*, when there is clear room for manoeuvre (2019: 864–5; compare with Ball *et al*., 2011 on England).

### International experiences of minoritization and marginalisation

Minoritization is a recurring theme in US studies of implementation. Their experiences help us categorise multiple modes of marginalisation in relation to race and migration, driven by witting/ unwitting action and explicit/implicit bias ( Farley *et al.,* 2019):

- *The social construction of students and parents*. Examples include: framing white students as ‘gifted’ and ‘high achieving’ and more deserving of merit-based education (or victims of equity initiatives) ( Turner & Spain, 2020: 796–7); treating non-white students as less intelligent ( Oakes *et al.,* 2005), more in need of special needs or remedial classes ( Thorius & Maxcy, 2015: 116–18), and having cultural or other learning ‘deficits’ that undermine them *and* disrupt white students ( Evans, 2009: 85; Felix & Trinidad, 2020: 480; Park *et al.,* 2012); and, describing migrant parents as unable to participate until they learn English ( Bertrand *et al.,* 2018: 8).
- *Maintaining or failing to challenge inequitable policies*. Examples include higher funding for schools and colleges with higher white populations ( Chu, 2019: 20; Felix & Fernandez Castro, 2018: 3) and tracking, which benefits white students disproportionately ( Oakes *et al.,* 2005; Turner & Spain, 2020).
- *Ignoring social determinants or ‘out of school’ factors* ( Bishop & Noguera, 2019).
- *Creating the illusion of equity with measures that exacerbate inequalities.* Promoting school choice policies while knowing that the rules restrict non-white access to sought-after schools ( Lenhoff, 2020)
- *Promoting initiatives to ignore race.* Examples include ‘colour blind’ or ‘equity for all’ initiatives ( Felix & Trinidad, 2020: 465–6).
- *Prioritising initiatives at the expense of racial or socio-economic equity.* Favouring measures to boost overall national performance at the expense of targeted measures ( Hemmer *et al.,* 2013).
- *Game playing and policy subversion.* School and college selection rules to restrict access ( Lenhoff, 2020) and improve metrics ( Li, 2019).

The wider international – primarily Global North – experience suggests that minoritization and marginalisation in relation to race, ethnicity, and migration is a routine impediment to equity strategies, albeit with some uncertainty about which policies would have the most impact. Schlicht-Schmälzle & Möller’s (2012: 1046) quantitative comparison of West European states finds a strong relationship between unequal educational attainment in mathematics (in PISA 2006) and immigration. However, curiously, a government’s *greater commitment* to ‘EU standards of good practice’ (‘educational programmes for migrant children and anti-discrimination policies’ to enable ‘equal participation in the education system and to gain the same achievements as their native counterparts’) is associated with *higher inequality* (2012: 1049; 1056). Further, the only countries that exhibit minimal inequalities are the (majoritarian) UK and Ireland, which challenges the argument that consensus democracies are ‘kinder’ and more conducive to equal outcomes (2012: 1056). Rather, they ‘enable the representation of large minorities in the political process’ (2012: 1060–1, countering Lijphart, 1999).

Further, multiple qualitative country studies describe the poor treatment of citizens in relation to immigration status or ethnicity, often while presenting the image of a more equitable system. Zilliacus *et al.* (2017: 232) contrast Finland’s (1) global reputation for education equity built on universalism and comprehensive schools, with (2) its historic ‘othering’ of immigrant populations, favouring national integration over global ‘social justice’. Only recently has it sought to ‘support cultural diversity and social justice as well as counter marginalisation and discrimination in education and society’.

Japan presents an unusual example of obliging foreign students to adapt. Tokunaga & Douthirt-Cohen (2012) relate its: (1) reputation for containing a homogeneous population, allowing its governments to present an image of classless egalitarianism and harmonious society, to (2) the ‘discriminatory and assimilative’ treatment of its over two million ‘registered foreigners’ (1.6% of the total population), including ‘the Koreans who were forcibly brought to Japan during the early part of the twentieth century as a source of cheap labor’ (2012: 321–2). Successive Japanese governments did not recognise or fund the ethnic high schools that developed from self-segregation (2012: 322). Indeed, the government only ceased to insist on a Japanese high school equivalency test for university entry in 2003, in response to international business concerns and the push to recognise international students (only if Japan has diplomatic ties with their home country, which excludes North Korea) (2012: 324).

Further, studies of Canadian provinces provide the strongest account of the symbolic and cynical use of multiculturalism for political gains and economic ends:

‘Multiculturalism has offered a safer, more palatable vocabulary for discussing uncomfortable subjects like racism and immigration, but in so doing, has blurred harsh realities about marginalisation and racialisation in this country and its education system ( George *et al.,* 2020: 170; 159).

Ontario and British Columbia policies contain three elements of ‘symbolic’ anti-racism: ‘1) the lack of robust education policy related to racial equity; 2) the construction of racism as an individual characteristic rather than a structural problem … and 3) the near-absence of race-related data collection’ ( George *et al.,* 2020: 159). Similarly, the combination of vague federal ambitions and Ontario government reluctance contributed to the veneer of multicultural policies. Policy documents accentuated multiculturalism’s contribution to global competitiveness, but hide ‘a Eurocentric curriculum, the streaming of at risk students into applied settings, and increased dropout rates among racialized students’ ( Segeren & Kutsyuruba, 2012: 24–5) and low teacher expectations for marginalised – Black and Latino - students ( Martino & Rezai-Rashti, 2013: 597–8). Toronto cultivated a reputation for ‘multi-cultural diversity’ without reversing its tendency to produce ‘growing inequality in income, health, access to services, housing, and transportation’ which exacerbate education inequalities ( Hamlin & Davies, 2016: 189; see also Gulson & Webb, 2013; Tamtik & Guenter, 2019: 41).

As in the US, many countries use ‘special needs’ categories to segregate immigrant and ethnic minority populations *.* Mainstreaming versus special needs debates have a clear racial and ethnic dimension when (1) some groups are more likely to be categorised as having learning disabilities or behavioural disorders, and (2) language and cultural barriers are listed as disabilities in countries such as ‘the USA, the UK, Australia, Canada, New Zealand, Germany, and Japan’ ( Chong, 2018: 502).

Alexiadou (2019: 427–8) identifies special needs categorisation as part of a collection of ‘techniques’ by national and subnational governments to segregate and discriminate against ‘the Roma minority in Europe’, exacerbating ‘high absenteeism and alienation’ and early school leaving. Three common measures are: (1) using linguistic, psychological, and pedagogic tests – and socioeconomic disadvantage – to describe proportionately more Roma children as in need of ‘preparation opportunities to enter mainstream education’; (2) providing low quality education in those classes, which limit progression to mainstream education; and/ or (3) boosting parental school choice to attend allegedly higher quality schools outside of a local area (which require resources in relation to access and transport). These measures allow policymakers in EU member states to avoid weakly-enforced EU legal sanctions, and subvert measures designed to promote ‘Roma inclusion in Europe’; their strategically-worded ‘on paper’ strategies - to fulfil their legal/ human rights obligation to promote ‘equality of outcomes’ – never leave the page (2019: 425–32; compare with McDermott *et al.,* 2014).

Further, ‘commonwealth’ country studies identify the marginalisation of *indigenous* populations in ways comparable to the US marginalisation of students of colour (e.g., Angus *et al.,* 2013; Loughland & Sriprakash, 2016; Molla & Gale, 2019; Morsy *et al*., 2014; Reid, 2017; Tobin *et al.,* 2016: 583 on Australia; Barker & Wood, 2019 on New Zealand; Tamtik & Guenter, 2019 on Canada).

## Discussion

### Connecting equity policy to public policy research

Policy process research helps interpret and compare experiences across sectors. In particular, Cairney *et al.* (2021: 23) argue that HiAP research lacks a realistic policymaking narrative, leading it to identify ‘unfulfilled expectations: why is there such a gap between evidence and policy, expected and actual levels of joined-up government, or strategy and implementation?’. Drawing on policymaking research, to ask how policy processes work, would help HiAP researchers manage their expectations on policy change, the use of evidence for policy, and the outcomes (2021: 23). Yet, HiAP research tends to engage in a circularity of enthusiasm and disappointment: (1) identifying the need for radical policy change, (2) promoting a new and ‘evidence based’ strategy to be adopted by each government, then (3) identifying implementation gaps, relating them to low political will, and expressing disillusionment with the politics of policymaking, before (4) restating the need for radical policy change (2021: 23).

In contrast, education studies identify the routine barriers to policy change, challenge rationalist top-down accounts of policy design, focus on the emergence of policy from multiple levels of government, and present more realistic narratives of policymaking. We focus on three key elements – the limits to (1) policy change, (2) processing evidence, and (3) policymaker control. We draw on Cairney (2020: 229–34) to summarise policymaking research, and the included and snowballed articles to relate these insights to education equity policy.

### The limits to policy change

Policymaking studies expect minor change in most cases and major change in few. They treat ‘policy’ as a collection of policy instruments – such as to redistribute resources, regulate behaviour, reform organisations, or share knowledge - whose overall impact is difficult to predict. Major change in one instrument does not necessarily cause change overall, and the meaning of proposed policy change in one issue or sector is unclear without relating it to policy change overall.

Similarly, education research shows that policy change is more apparent on paper than practice. Country governments and international organisations express strong support for a multi-faceted approach to improving education equity, but most studies contrast it with limited change in practice. One indicator of lip-service is when policymakers describe a commitment to equity without saying which policy instruments they will use (e.g., exhortation, regulating schools, or reforming tax and spending for schools) ( Louis *et al*., 2008: 571). At the same time, tracking and other inequitable practices endure despite widespread criticism from professional groups and the OECD. Further, inequitable policy outcomes endure despite the signal by governments that they will change, such as the ‘achievement gap’ related strongly to minoritization and the social determinants of education (e.g., Gorard, 2018: chapter three). Overall, we find policies designed *ostensibly* to promote equity, but equity is a low priority overshadowed or undermined by other aims.

### The limits to processing evidence

Policymaking is not a rationalist ‘evidence based’ process. Rather, policymakers must find ways to ignore almost all information to make choices, and their choices do not solve the problems they address ( Cairney, 2016; Cairney, 2021; compare with Gorard, 2018; Wiseman, 2010). To deal with their ‘bounded rationality’ ( Simon, 1976), they rely on cognition, emotion, beliefs, and standard operating procedures to interpret and prioritise information. They rely on trusted sources to reduce uncertainty. They exercise power to reduce policy ambiguity: focusing attention on one of many possible ways to understand a problem. Policy theories use these insights to explain key policymaker responses, including:

1. *Paying more attention to some problems and solutions than others.*

Policymakers process information disproportionately: they pay high attention to some issues and ignore most others, and favour some problem definitions while neglecting others ( Baumgartner & Jones, 2009; Koski & Workman, 2018). Dominant beliefs within a policy network influence their perceptions of the technical and political feasibility of policy solutions. Indeed, policymakers only pay sustained attention to problems for which there is a feasible solution ( Kingdon, 1995). For example, a social justice approach to education equity receives lower attention than aims related to access, efficiency, quality, performance, and economic competitiveness. In some cases, policymakers treat educational inequity as a ‘wicked’ problem that defies feasible solutions ( Farley *et al.,* 2019; Reid, 2017: 88, citing Rittel & Webber, 1973). Or, governments promote greater equity as a by-product of the policies they favour.

2. *Forming coalitions of like-minded actors and competing with other coalitions.*

The Advocacy Coalition Framework (ACF) suggests that people enter politics to turn their beliefs into policy, forming coalitions with actors who share their beliefs, and using beliefs to interpret and learn from policy-relevant evidence ( Sabatier & Weible, 2007). In highly polarised issues, coalition members romanticise their own cause while demonising their opponents ( Sabatier *et al.,* 1987). In less polarised issues, there is scope for common ground and for experts to facilitate policy-oriented learning ( Ingold & Gschwend, 2014).

DeBray *et al*.’s (2014: 175) study of New Orleans uses the ACF to explain the competitive use of evidence to assess how equitable are ‘incentivist’ programmes such as voucher schemes, school choice, and charter schools. There is some focus on depoliticising policy – via a rhetorical language regarding ‘scientifically based research’, ‘what works’, and ‘data-driven decision-making’ – but also low policymaker demand for research, and low research capacity. There is high contestation to evaluate policies, in a polarized ‘political landscape of research … characterized by mistrust’ (2014: 182; 195). One coalition describes incentives as successful (based on poor quality research produced by the actors who benefit) and most policymakers want evidence of their success to bolster their beliefs. The other coalition declares failure, but few organisations have the resources to challenge policymaker bias or the biased evidence (2014: 196). What appears to be an ‘evidence based’ process, to establish the equitable impact of incentivist schemes, is a political process to sell their value.

Using social networks analysis, Kretchmar *et al.* (2016: 423) identify a similar dynamic within multiple policy networks. Organisations such as Teach for America provide 0.2% of teachers (5,000 per year, from a short training course) but have disproportionate network influence: working with large philanthropic organisations, ‘credential providers’, ‘market suppliers’ and ‘legislative supporters’ to support education ‘privatization’ and market reforms, while relating inequity to poor teaching or a lack of teacher autonomy and innovation. Such coalitions operate within networks that ‘act as “shadow states,” in which unelected, decentralized bodies exercise profound influence on public policy without democratic oversight’ (2018: 431).

3. *Emulating other governments, or relying on international organisations*

Studies of policy diffusion and transfer suggest that some governments respond to bounded rationality by emulating others without gathering evidence, because they (a) *assume* that the other government changed policy successfully, (b) feel pressure to keep up with domestic or international norms, or (c) are persuaded by ‘policy entrepreneurs’ (including wealthy corporations or philanthropic organisation) of the benefits of importing a policy ( Berry & Berry, 2018; Bulmer *et al*., 2007; Cairney *et al*., 2021; Dolowitz & Marsh, 1996).

Multiple education studies highlight the role of certain countries as beacons for change despite limited evidence for success (e.g., Hamlin & Davies, 2016 on Canada), or as contributing to international organisation pressure to conform to a global agenda. Many draw on Rizvi & Lingard (2010: 80–91; 121–2) to describe how governments import ideas (on human capital and the global knowledge economy), techniques (NPM), and programmes (the privatization of education and promotion of school choice), without clear evidence that they improve outcomes.

Tarlau & Moeller (2020: 343) identify the disproportionate influence of the Lehman foundation (funded by ‘the richest man in Brazil’) when the Brazilian government emulated the US’ Common Core initiative in 2017. This experience is indicative of the importance of ‘educational policy transfers across national borders … occurring through networks of private and corporate actors’; donor groups open the door for policy changes that allow private companies to profit from market reforms (2020: 357–8). Organisations turn the complexity of education equity into a simple technical concern about common standards, as part of a focus on performance management and meritocracy which ‘ignores the structural forms of educational marginalization that individuals and communities face if they are poor, Black, mixed race, or indigenous’ (2020: 360).

4. *Socially constructing target populations*

Social construction and policy design (SCPD) studies show how policymakers use social stereotypes to describe groups as deserving or undeserving of government benefits ( Schneider & Ingram, 1997). It can be a strategic move by politicians seeking popularity and their preferred policies, or an emotional reaction to their beliefs ( Schneider *et al*., 2014: 106). For example, as described above, white students are often portrayed as more deserving of merit-based education (or victims of equity initiatives), with students of colour, immigrant, or indigenous populations portrayed as in need of remedial classes ( Bertrand *et al*., 2018; Brezicha & Hopkins, 2016; Evans, 2009: 85; Halverson & Plecki, 2015). These statements contribute to financial allocations and tracking. Few studies highlight successful attempts to portray minoritized populations as more worthy of government benefits, even as victims of unequal processes.

5. *Presenting an image of ‘evidence-based policymaking’ to depoliticise policy*

Policymakers tend not to reflect publicly on their cognitive and organisational limits. Many governments present the opposite story, using slogans like ‘evidence-based policymaking’ to present an image of governing competence, and depoliticising issues by describing them as technical and amenable to scientific solutions ( Cairney, 2016). Such discursive strategies may be part of a larger package of depoliticization measures to question the role of the state and pull back from problems such as inequity ( Bacchi, 2009; Stone, 2012; Wood, 2016: 523).

This political attempt to depoliticise policymaking and exclude non-expert voices is a central theme in education equity research. It highlights a tendency for governments to use the language of rationalism to defend policy, generally at the expense of social justice frames ( Halverson & Plecki, 2015: 46–50; Klees & Qargha, 2014; Louis *et al.,* 2008: 566). Critical policy analysis is a means to challenge this story ( Allbright *et al.,* 2019: 178; Felix & Fernandez Castro, 2018: 10; Marshall & McCarthy, 2002: 481; Thorius & Maxcy, 2015: 117). Studies highlight the intentional and unintentional consequences of this dominant framing of policymaking, such as the lack of direct and sustained focus on:

- *minoritization* ( Chu, 2019; Felix & Fernandez Castro, 2018; George *et al.,* 2020; Morsy *et al*., 2014; Tamtik & Guenter, 2019)
- *socioeconomic background* ( Clarke, 2012; Taylor, 2004)
- *gender* ( Gill, 2005; Marshall & McCarthy, 2002)
- *inequalities of participation* ( Porras, 2019; Spreen & Vally, 2010)
- *inequalities in resources* ( Fallon & Paquette, 2009; Hanna & Gimbert, 2011; Penney, 2017).

Some studies highlight the dilemmas of operating within this context: advocates for racial equality may object to neoliberalism but know that market-based tools may be the only means to achieve progress ( Gulson & Webb, 2013: 175–8).

This theme is also central to the snowballed literature, some of which presents a story of post-war ‘rationalist’ policymaking in which policymakers and analysts believed that the major expansion of scientific analytical techniques, and highly centralised policymaking, could help solve major policy problems (see Cairney, 2021: 35–6, drawing on Radin, 2019; Brans *et al*., 2017).

Rizvi & Lingard (2010: 2; see also Ball, 1998) describe a recent reduction in faith in *scientific policy analysis* (coupled with the rise in attention to critical policy analysis), *centralized policymaking* (and rise in globalization and multi-level policymaking), and *the sense that state intervention would solve major policy problems* (in favour of market reforms). These trends underpinned a global shift in education policy, with a major expansion of education capacity accompanied by ‘market solutions’ fostered by governments that were ‘unable or unwilling’ to pay for it (2010: 3). Rizvi & Lingard (2010: 3; 54–6) seek to explain the ‘global dominance of the neoliberal policy paradigm’ and ‘how it might be unravelling in the current global economic crisis’, using critical policy analysis to ‘forge a different, more just and democratic globalization that implies a broader conception of education’s purposes’ (see also Thomson, 2013).

Nevertheless, an appeal to rationalism via quantification – ‘governing by numbers’ - remains a powerful tool, associated with ‘the governing effect that numbers have in bringing together national and organisational storylines on the status of education’ ( Grek, 2020: 140–1; 146; see also Lawn, 2011; Lingard, 2011; Ozga *et al.,* 2011; Ozga, 2017; Spillane, 2012).

### The limits to policymaker control

Policymakers act in a complex policymaking system or environment of which they have limited knowledge and less control ( Cairney *et al*., 2019). While central governments are powerful actors, policy outcomes emerge from their environments containing:

- Many policymakers and influencers spread across multiple levels of government (actors).
- Multiple venues for authoritative choice, each with their own informal and formal rules (institutions).
- Relationships between the actors responsible for making policy and those who influence and deliver it (networks).
- Dominant beliefs and assumptions about the policy problem (ideas).
- The socio-economic factors and events that influence policymakers and are out of their control (policy context or conditions).

Policy studies describe these dynamics in multiple ways. For example, Kingdon (1995) is popular in HiAP studies because ‘multiple streams analysis’ offers hope for major policy change, prompted by ‘policy entrepreneurs’ ( Cairney, 2018), during a ‘window of opportunity’ in which three ‘streams’ come together:

1. ‘Problem stream: there is heightened attention to a policy problem.
2. Policy stream: a technically and politically feasible solution is available.
3. Politics stream: policymakers have the motive and opportunity to select it’ ( Cairney *et al*., 2021: 26).

Yet, these opportunities are rare and unpredictable, and not in the gift of entrepreneurs or policymakers. Nor does the choice to select a policy solution determine policy outcomes, particularly when the choice is a vague ambition such as equity.

Further, policy studies highlight ‘path dependence’ ( Pierson, 2000) associated with ‘policy feedback’ ( Mettler & SoRelle, 2018), when choices made in the past inform current institutions. For example, well-established political system rules help reproduce the (a) unequal distribution of ‘benefits and burdens across racial groups’ and (b) relative distribution of resources towards supportive (e.g., education) and punitive (e.g., prisons) policies ( Michener, 2019: 7). Further, levels of policymaking centralisation or decentralisation can challenge or exacerbate their inequitable effects (2019: 11).

Similarly, complexity studies highlight a tendency towards path dependence *and* for policy outcomes to ‘emerge’ locally in the absence of central government control. Frustration with emergent outcomes often drives governments to try to reassert control via NPM ( Geyer, 2012; Weaver-Hightower, 2008). Yet, they do so in vain, and produce unintended consequences. Further, studies of multi-level governance and bottom-up policymaking show how policy changes as it is implemented, such as when its delivery requires cooperation between many governmental and non-governmental actors ( Cairney, 2020: 106). Therefore, while there may be pressure to transfer policy, path dependence influences how actors make sense of policies in new contexts.

These themes are prevalent in education research ( DeBray *et al*., 2014; Gilead, 2019; Louis *et al.,* 2008; Marshall, 2000; Pinheiro *et al*., 2016; Rorrer *et al*., 2008; Tokunaga & Douthirt-Cohen, 2012; Turner & Spain, 2020; Varjo *et al.,* 2018; Yazan, 2014). Further, comparable concepts, such as a ‘zone of mediation’, capture similar dynamics in relation to limited policy change ( Brezicha & Hopkins, 2016; Thorius & Maxcy, 2015: 119; Trujillo, 2012).

In particular, our discussion of implementation highlights complicated relationships between levels of government. On the one hand, local school and district leaders have discretion to make sense of policy as they deliver, and challenge top-down agendas ( Molla & Gale, 2019; Wang, 2018). Therefore, we do not understand policy continuity or change unless we understand how practitioners make sense of it ( Feldman & Winchester, 2015; Hemmer *et al.,* 2013; Kornhaber *et al.,* 2017; Trujillo, 2012; see also Spillane *et al*., 2012) or their resources to deliver ( Meyer *et al.,* 2018). On the other hand, their actions take place in a wider context of multi-level policymaking, in which neoliberal global and national agendas constrain their discretion, while local community or parental opposition limits their role as ‘change agents’.

Further, the snowballed texts suggest that, while neoliberal global and national agendas are pervasive, their impact varies markedly across political systems and time ( Apple, 2001; Rizvi & Lingard, 2010: 42). ‘Generic solutions’ are translated and transformed in local contexts ( Ball, 1998: 126–7). Steiner-Khamsi’s (2014: 154) description of borrowing from PISA league leaders suggests that policymakers ‘only emulate the system features of league leaders if it fits their own domestic policy agenda’, and borrowing comes with the need to translate into local contexts ( Steiner-Khamsi, 2012: 4). The ‘window of opportunity’ to borrow varies markedly, the adoption of the initiatives across the globe can be separated by over a decade, and some countries rely on international organisation funding for policy change ( Steiner-Khamsi, 2006: 674). Rizvi (2016: 5) describes similar variations in the ‘privatization of education’, which can include public-private cost sharing, quasi-markets, voucher schemes, and non-state services (compare with Patrinos *et al.,* 2009).

Included studies suggest that, in many Global South countries, neoliberal influence goes beyond ideational dominance; international organisations such as the World Bank insist on particular actions when setting conditions for financial support (e.g., De Lisle, 2012; Singh, 2011; see also Bekisizwe & Lubienski, 2017). In countries like the US, which helps to drive this international agenda, the dynamic of performance management and focus on access to schools accompanies a narrow concern with equity via test scores. In Nordic countries, the experience is mixed: Sweden highlights a greater tendency to seek ‘recentralisation’ and the profound impact of quasi-market measures on unequal access to schools, Norway demonstrates continuous tensions between decentralised delivery and national accountability, but Finland highlights the ability to incorporate global agendas into existing rules and norms ( Camphuijsen *et al*., 2020: 12–14; Pettersson *et al.,* 2017: 732; Varjo *et al.,* 2018; Wahlström, 2014). Within this spectrum are countries like Australia, which seems relatively conducive to neoliberal reforms ( Loughland & Thompson, 2015), and Canada, importing US-style policies more selectively *and* selling policy solutions to many other countries ( Hamlin & Davies, 2016; Mindzak, 2015). There are also mixed dynamics *within* the US: Baker (2019) identifies the ‘policy diffusion’ of bans on affirmative action in US states, but their adoption and meaning varies according to political cultures and the perceived level of ‘racial threat’ in each state.

### Limitations

No search or review is comprehensive, and it is possible that a large series of searches for specific organisations (such as UNESCO) would have yielded more results comparable to our HiAP review. However, we used a relatively general keywork search, combined with manual inclusion/exclusion processes, to immerse ourselves in the education field, and identify the main foci of education equity researchers, to avoid biased searches through a health equity lens. We also used snowballing when it became clear that education research has a relative focus on key texts/ approaches rather than key international organisations or strategies. The more pressing limitation is a bias in research towards Global North experiences. As such, while the Results and Discussion sections identify the clear implications - of limited policy progress towards education equity – for policymaking and practice, their applicability is by no means universal.

## Conclusion

We began by noting that COVID-19 had a major global impact on education, prompting attention to its unequal effects and presenting some impetus to reboot equity strategies. In HiAP studies, it is common to relate such attention to a ‘window of opportunity’ for major policy change. However, high attention to the problem of inequalities is only one part of the story, since the definition of that problem and the feasibility of solutions is highly contested, and the motive and opportunity for policymakers to act may come and go.

In that context, our review suggests that our understanding of future global and domestic education equity policies is limited without establishing how policymakers currently make sense of equity. For example, global policy agendas suggest that there is high support for equity initiatives, but defined in relation to education’s role in the economy, and pursued in relation to equality of access to public services. This approach tends to dominate discussions and receive support from key international organisations and countries, at the expense of the wider focus on social justice, or social determinants of educational outcomes, supported by most articles in our review. Therefore, we would expect a restatement of international support to reboot programmes to improve access to schools, despite a general warning in review articles that ‘equal’ access does not secure equity (and often exacerbates inequalities).

We describe education equity researchers as the meta-narrators of cautionary tales of education inequity. They employ critical policy analysis to challenge the dominant stories of education that hinder meaningful equity policies (see Jones *et al.,* 2014 on constructing narratives containing a setting, plot, characters, and moral).

First, many describe common settings: the inequalities that endure despite global and domestic equity commitments; and, the multi-level nature of the governance of education. A small number of international organisations and countries are key influencers of a global neoliberal agenda *and* there is discretion to influence policy at local and school levels. Further, some studies relate the lack of progress to the malign influence of one or more levels, such as global and central government agendas undermining local change, or local actors disrupting central initiatives.

Second, studies describe similar plots. Many describe stymied progress on equity caused by the negative impacts of neoliberalism and NPM: both undermine equity by equating it with narrow definitions of equal access to well-performing schools and test-based attainment outcomes, and they take attention from social justice to focus on economic competitiveness. Many describe policymakers using a generic focus on equity as a veneer, to ignore and reproduce inequalities in relation to minoritization. Or, equity is a ‘wicked’ issue that defies simple solutions. Many plots involve a contrast between agent-focused narratives that emphasise hopefulness (e.g., among ‘change agents’) and systemic or structural narratives that emphasise helplessness.

Third, they present common ideas about characters. In global narratives, researchers challenge the story by international organisations that they are the *heroes* providing funding backed by crucial instructions to make educations systems and economies competitive. Education articles portray neoliberal international organisations and central governments as the *villains*: narrowing equity to simplistic measures of performance at the expense of more meaningful outcomes, to the detriment to a much-needed focus on social justice. At a national and local level, they criticise the dominant stories of equity within key countries, such as the US, that continue to reproduce highly unequal outcomes while projecting a sense of progress. The most vividly told story is of white parents, who portray their ‘gifted’ children as most deserving of advantage in the school system, and therefore the victims of attempts to widen access or redistribute scarce resources (high quality classes and teachers). Rather, these parents are the villains standing – sometimes unintentionally, but mostly intentionally - in the way of progress. The only point of uncertainty regards the role of local and school leaders. In some cases, they are the *initially* heroic figures, able to find ways to disrupt a damaging national agenda and become the ‘change agents’ that shift well-established rules and norms before being thwarted by community and parental opposition. In others, they are *perhaps*-unintentional villains who reproduce racialised norms regarding which students are ‘gifted’ and worthy of investment versus which students need remedial classes or disrupt other learners.

Fourth, the moral of the story is mostly clear. Almost all studies criticise the damaging impact of neoliberal definitions of equity and the performance management and quasi-market techniques that support it. They are sold as equity measures but actually exacerbate inequalities. As such, the moral is to focus our efforts elsewhere: on social justice, the social and economic determinants of education, and the need to address head-on the association between inequalities and minoritized populations (to challenge 'equity for all' messages). However, it is difficult to pinpoint the *source* of much-needed change. In some cases, strong direction from central governments is necessary to overcome obstacles to change. In others, only bottom-up action by local and school leaders will induce change.

In other words, unlike our study of HiAP ( Cairney *et al.*, 2021), we do not find a common (top-down) strategic vision and ‘playbook’ taken forward – often in vain - by international organisations, domestic governments, and academic research. Rather, education research recognises the contested nature of equity policy and the need to discuss that contestation. It also highlights policymaking complexity and the need to give proper acknowledgement to the bottom-up processes that constrain or facilitate progress. This approach does not *solve* the problem of education inequity, but it allows academics and practitioners to reflect on the dilemmas that accompany equity policies. As such, it has a lot to offer HiAP’s agenda on intersectoral action (see Cairney *et al.*, 2022).

## Data availability

### Underlying data

All data underlying the results are available as part of the article and no additional source data are required.

### Extended data

Open Science Framework. Qualitative systematic review of lessons from education policymaking. https://doi.org/10.17605/OSF.IO/BYN98. ( Cairney & Kippin, 2021).

This project contains the following extended data

- structured bibliography to accompany this review.
- Search protocol.

Data are available under the terms of the Creative Commons Zero "No rights reserved" data waiver (CC0 1.0 Public domain dedication).

### Reporting guidelines

OSF. PRISMA checklist for ‘The future of educational equity in a COVID-19 world: a qualitative systematic review of lessons from education policymaking’. https://doi.org/10.17605/OSF.IO/BYN98.

Data are available under the terms of the Creative Commons Zero "No rights reserved" data waiver (CC0 1.0 Public domain dedication).
