# Supplementary material for: The future of education equity policy in a COVID-19 world: a qualitative systematic review of lessons from education policymaking
Source: Open Res Eur. 2022 Jan 13;1:78. Originally published 2021 Jul 8. [Version 2] doi: 10.12688/openreseurope.13834.2 (PMC10445953; doi:10.12688/openreseurope.13834.2)
Supplement: Peer review history

# Peer review: Reviewer response for version 2

**DOI:** 10.21956/openreseurope.15549.r28357

**Published:** 2022-05-12

**Authors:** Elizabeth Farley-Ripple

**Affiliations:** 1 School of Education, University of Delaware, Newark, DE, USA

I appreciate the authors' attention to the issues raised in my first set of comments. I have re-read the manuscript and amend my report to recommend status to approved.

Are sufficient details of the methods and analysis provided to allow replication by others?

Partly

Are the rationale for, and objectives of, the Systematic Review clearly stated?

Partly

Is the statistical analysis and its interpretation appropriate?

Yes

Are the conclusions drawn adequately supported by the results presented in the review?

Yes

Reviewer Expertise:

NA

I confirm that I have read this submission and believe that I have an appropriate level of expertise to confirm that it is of an acceptable scientific standard.

# Peer review: Reviewer response for version 2

**DOI:** 10.21956/openreseurope.15549.r28356

**Published:** 2022-02-17

**Authors:** Christina Hadjisoteriou

**Affiliations:** 1 School of Education, University of Nicosia, Nicosia, Cyprus

I have now read the updated version of the article. I am happy with the changes made. I amend the status to approved.

Are sufficient details of the methods and analysis provided to allow replication by others?

Partly

Are the rationale for, and objectives of, the Systematic Review clearly stated?

Yes

Is the statistical analysis and its interpretation appropriate?

Partly

Are the conclusions drawn adequately supported by the results presented in the review?

Partly

Reviewer Expertise:

NA

I confirm that I have read this submission and believe that I have an appropriate level of expertise to confirm that it is of an acceptable scientific standard.

# Peer review: Reviewer response for version 1

**DOI:** 10.21956/openreseurope.14915.r27869

**Published:** 2021-12-01

**Authors:** Christina Hadjisoteriou

**Affiliations:** 1 School of Education, University of Nicosia, Nicosia, Cyprus

**Are the rationale for and objectives of the systematic review clearly stated?**

 The authors have provided a thorough and well-sounded rationale for their systemic review. To begin with, the authors explain that their review draws upon their previously carried out review in the ‘Health in All Policies’ (HiAP) field. However, it would have been helpful for the reader, if the authors had provided a brief summary of their HiAP review, so that we could better understand the linkage between their first and second review. Furthermore, the authors have brought to the surface the academic debate with regards to the contested nature of educational policies for equity across a neo-liberal world that claims to seek out equity in education. Lastly, they added to their rationale by making reference to contemporary phenomena such as Covid-19 that intensify the urge to recognise the continuously-evolving character of equity, while creating new conditions to be taken into consideration in pedagogy and teaching that should be seriously taken into consideration in the making of equity education policies.

 Taking into consideration their rationale, the authors set out the umbrella question that led their review: ‘ *How does education equity research use policy theory to understand policymaking?’, *accompanied by the following series of sub-questions:

- How many studies provide a non-trivial reference to policymaking concepts or theories?
- How do these studies describe policymaking?
- How do these studies describe the ‘mechanisms’ of policy change that are vital to equity strategies?
- What transferable lessons do these studies provide? For example, what lessons for other governments do case studies provide?
- How do these studies relate educational equity to concepts such as spatial justice?

Thereafter, the authors moved to the delineation of the objectives underlying their review. Namely, they stated the following five areas of examination:

1. Establish if there is a coherent international education policy agenda to which each article contributes.
2. Analyse the contested definition of equity: what exactly does it mean?
3. Explore critiques of ‘neoliberal’ approaches to education equity.
4. Compare top-down and bottom-up perspectives on policymaking complexity.
5. Identify the impact of minoritization and marginalization.

Arguably, the need to carry out a systemic review of the ways past literature and research has ‘treated’ the equity issue in the field of education policy is imperative as such review could potentially contribute to inform policy-making and the building of education policies that cultivate social justice and social cohesion, and supporting the development of inclusive societies in and out of schools. Nonetheless, there are some important issues that the authors did not take into consideration in their article. For example, what do the authors mean by ‘policy theory’, ‘spatial justice’, ‘equity’ etc. Addressing these concepts would have added to their rationale and objectives.

**Is the statistical analysis and its interpretation appropriate?**

 The question is not applicable for the aforementioned systematic review, as no statistical analysis was carried out, nor it was appropriate for the purposes of meeting the objectives of this review.

 The authors are indeed very descriptive of their findings, and I do believe that they did a great work in bringing all this research together. Although their review was rather ambitious in terms of workload and degree of difficulty, they managed to provide in-depth and thorough description of the tensions and ambiguities that exist among and between education policies for equity within and across nation states and international organisations. I do believe that we do need more of such kind of detailed reviews that encompass multiple levels of analysis (the national and the international, the local and the focal, the top-down and the bottom-up).

 However, the authors did not build a strong theoretical framework within which they could have set in their review. Such framework could have guided the interpretation of their findings. To do so, the authors could had unpacked ‘policy theory’ - which is the main ax of analysis that is stated in their umbrella question - by providing not only a working definition of policy theory for their review, but by building the ‘conceptual filter’ that they could use as a lens to carry out their review. The authors also fell short to adequately unpack the notions of ‘spatial justice’, ‘equity’ (although they provide a very short definition of vertical and horizontal equity), and ‘social justice’, and their interrelation. Thus, my concern is that while the authors have masterfully provided the reader with very clear objectives underlying their work, as they did not delineate the theoretical and conceptual underpinnings of their review, the reader is left wondering how the authors reached out to the specific findings and conclusions from their review.

**Are sufficient details of the methods and analysis provided to allow replication by others?**

 The authors clearly explained the sampling process regarding the selection of the articles to be included in their review. They provided the reader with extensive information on the eligibility criteria underlying their selection processes. The authors also provided information with regards to the databases that were used, the search terms, and the geographical attributions of the research selected to be included in their review. 

 Nonetheless, the authors are missing the description of the coding stage, that follows the selection stage in a systematic review. The coding stage allows the authors to record information from each study that allows them to answer their review questions. To carry out their systematic analysis, the authors could had followed Newman and Gough’s (2019) coding strategy, which includes the following: ‘(i) details of the studies to enable mapping of what research has been undertaken; (ii) how the research was undertaken to allow assessment of the quality and relevance of the studies in addressing the review question; (iii) the results of each study so that these can be synthesised to answer the review question’ (p. 12).

 Furthermore, the authors explained that they had used an inductive qualitative approach to reach out to themes so as to respond to the initial questions that guided their review. However, the authors did not provide the reader with adequate information regarding the synthesis stage: how did they set out to search for patterns and results by identifying commonalities and disparities across the selected studies? The authors could had referred to data analysis techniques adopted from primary qualitative research, such as re-reading, descriptive and analytical coding, theme development, constant comparison, and iteration with theory. The authors could have had also explored whether data and results could be configured in different ways, while integrating them to address their review questions. Last but not least, the authors did not reflect on issues regarding the quality of their synthesis. For example, Carroll and Booth (2015) suggest that what we should look at in qualitative research is the authenticity of each selected study in reflecting on the meaning of the data. 

   

**Are the conclusions drawn adequately supported by the results presented in the review?**

 When it comes to carrying out a systematic review, Hart (2018) explains that such a process sets out to synthesise substantive findings, while reflecting on the theoretical and methodological contributions of previous research in a specific field. For the purposes of carrying out a systematic review, authors should draw upon a broadly configurative synthesis logic aiming to ‘investigate research questions about meaning and interpretation to explore and develop theory’ (Newman & Gough, 2019, p. 4-5). As I have indicated above, as the authors did not provide a theoretical framework, they did not discuss their findings vis-à-vis a clearly articulated framework (beyond their rather vague references to ‘policy theory’). Therefore, the authors did not provide a thorough section focusing on the conclusions of the review with regards to the development of theory.

 Beyond developing theory, although the analysis seems to be well written, the conclusion section is too short and rather ‘light’ in terms of reflections: What are the implications of this systematic review for equity education policies in terms of development and implementation? Does this review bear wider implications for other reviews to be carried out in the field? What are the lessons to be learnt?

Are sufficient details of the methods and analysis provided to allow replication by others?

Partly

Are the rationale for, and objectives of, the Systematic Review clearly stated?

Yes

Is the statistical analysis and its interpretation appropriate?

Partly

Are the conclusions drawn adequately supported by the results presented in the review?

Partly

Reviewer Expertise:

NA

I confirm that I have read this submission and believe that I have an appropriate level of expertise to confirm that it is of an acceptable scientific standard, however I have significant reservations, as outlined above.

## References

[1] : Doing a Literature Review: Releasing the Research Imagination. *SAGE.* .2018;

[2] : Systematic reviews in educational research: Methodology, perspectives and application. In: O. Zawacki-Richter, M. Kerres, S. Bedenlier, M. Bond, K. Buntins (Eds) Systematic Reviews in Educational Research: Systematic Reviews in Educational Research. *Springer VS* .2019;3-22

[3] : Quality assessment of qualitative evidence for systematic review and synthesis: Is it meaningful, and if so, how should it be performed?. *Res Synth Methods* .2015;6(2) : 10.1002/jrsm.1128 149-54 10.1002/jrsm.1128 26099483

# Response

**Published:** 2021-12-22

**Authors:** Paul Cairney

**Affiliations:** University of Stirling, UK

Paul Cairney and Sean Kippin 22.12.21 p.a.cairney@stir.ac.uk Notes on the revision of ‘The future of education equity policy in a COVID-19 world: a qualitative systematic review of lessons from education policymaking’ https://open-research-europe.ec.europa.eu/articles/1-78/v1   We thank both reviewers for their thorough and constructive reviews, which helped us to improve our review. We summarise how the article has changed in response, then provide a full description of responses to each reviewer. Summary of changes:

1. Introduction: we (a) clarified the role of mainstream policy theory and (b) summarised the health equity strategy (HiAP) used as a comparison with education equity policy (pp3-6).
2. Methods: we clarified the (a) role of the IMAJINE project’s research questions and approach, (b) importance of immersion and induction, (c) coding, and (b) approach to analysis and synthesis (pp6-9).
3. Results: we altered subheadings and formatting to address the lack of clarity of some parts of the presentation. We edited this section to reduce the word count (to accommodate changes prompted by each reviewer) (pp10-35).
4. Conclusion: we reorganized and improved the discussion on policy and research implications (pp39-40).

**Dr Christina Hadjisoteriou**

1. *Provide a brief summary of the HiAP review, to help understand the linkage*.
  We add a further paragraph on HiAP in the introduction, immediately before a set of headings that identify key comparisons between HiAP and education equity studies. These comparisons help to provide the structure for the initial analysis of new findings in Results.
2. *Clarify what we mean by ‘policy theory’, ‘spatial justice’, ‘equity’ etc. in rationale and objectives*
3. *Show how our understanding of policy theory acts as the conceptual filter to carry out our review*.
  We make it clearer in the Method section that we use a separate text to analyse ‘spatial justice’ (but include the spatial justice question there to describe the wider IMAJINE project). In the Introduction, we clarify that there is no single definition of equity in political systems (beyond the general statement that inequity tends to mean unfair inequality, and the meaning of fairness is contested). Rather, our focus is on the contestation to define it (in ***2. Analyse the contested definition of equity: what exactly does it mean?*)**. In the Introduction, we summarise the three key insights from mainstream policy theories that guide our understanding of policy and policymaking (and which help to structure our Results section). The new text appears in this paragraph: **‘Therefore, advocates and researchers of education policy reforms need to draw on policymaking research to understand the processes that constrain or facilitate equity-focused initiatives. In particular, a collection of ‘mainstream’ policy theories identifies three ever-present dynamics (Cairney (2020: 229–34)). First, most policy change is minor, and major policy changes are rare. Second, policymaking is not a rationalist ‘evidence based’ process. Rather, policymakers deal with ‘bounded rationality’ (Simon, 1976) by seeking ways to ignore almost all information to make choices. Third, they operate in a complex policymaking environment of which they have limited knowledge and control. Without using these insights to underpin analysis, equity policy research may tell an incomplete story of limited progress and address ineffectively the problem it seeks to solve. In that context, the guiding question of our review is: *How does education equity research use policy theory to understand policymaking?’* These three insights provide the structure for analysis in the Discussion section. In methods, we (a) clarify the meaning of mainstream policy theories, but also (b) show how and why we do not seek to limit the search only to articles that engage with the mainstream (put simply, it would narrow the inclusion criteria too much, producing an unhelpfully small sample of articles to analyse).’**
4. *Provide an explicit discussion of the coding stage*.
  We now clarify the coding stage and note how we consolidated the list of categories in this review.
5. *Provide more explanation of the synthesis stage*.
  We clarify that our approach follows Sandelowski and Barroso’s (2007: xv) advice to foster respect for each author’s methods and aims while seeking to answer our guiding questions. We describe our approach in a new paragraph in Method [beginning **‘Fifth, we used an inductive qualitative approach to analyse each text, generate themes (Results), and relate them to policy theory insights (Discussion)’**].

# Peer review: Reviewer response for version 1

**DOI:** 10.21956/openreseurope.14915.r27363

**Published:** 2021-08-31

**Authors:** Elizabeth Farley-Ripple

**Affiliations:** 1 School of Education, University of Delaware, Newark, DE, USA

**Are the rationale for and objectives of the systematic review clearly stated?**

 The manuscript has a very clearly defined and well articulated purpose for the review, adding to their prior work related to HiAP and providing insights into the literature on an incredibly salient policy issue globally. The authors situate this work in the COVID-era, offering an opportunity to learn from prior work as we collectively prepare for a more equity-centered recovery. The authors thus equally anchor the project in prior literature (on equity and policymaking) as well as in the current global context. 

 The authors also note specific objectives for the review, which are largely aligned with their rationale and met in the results and discussion. However, there are a few inconsistencies worth noting and which may be (in my opinion) revised simply. 

 First, the authors offer the central question: *How does education equity research use policy theory to understand policymaking?* However, they do not actually appear to tackle how the research uses policy theory. In some cases this is true (e.g. the use of zone of mediation) but a better description of the manuscript is that the authors provide a) a rich and well written synthesis of the educational equity policy literature organized around 5 issues (1. Establish if there is a coherent international education policy agenda to which each article contributes; 2. Analyse the contested definition of equity: what exactly does it mean?; 3. Explore critiques of ‘neoliberal’ approaches to education equity; 4. Compare top-down and bottom-up perspectives on policymaking complexity; 5. Identify the impact of minoritization and marginalization) followed by b) their analysis of that literature in terms of policy theory as presented in the discussion. If I am understanding and stating their intent correctly, I would recommend the authors revise the central question to something like: How can we use policy theory to understand policymaking in educational equity research (or something like that)?

 Second, the authors suggest subquestions for their central question:

1. How many studies provide a non-trivial reference to policymaking concepts or theories?
2. How do these studies describe policymaking?
3. How do these studies describe the ‘mechanisms’ of policy change that are vital to equity strategies (although Cairney *et al*., 2021 show that very few studies answer this question)?
4. What transferable lessons do these studies provide? For example, what lessons for other governments do case studies provide?
5. How do these studies relate educational equity to concepts such as spatial justice? (we answer question 5 in Cairney *et al*., 2022).

As a reader, I’m not sure that the answers to these questions actually guide their inquiry and presentation of results. For example, subquestion 1 appears to be answered as part of the methods, but that seems more like the process of selection than an analysis of the extent to which educational equity policy research attends to particular concepts and theories. An answer to that question, in my opinion, might include a discussion of the types of theories raised in various studies, quantified and elaborated on by example. Rather, that was not a strong feature of the manuscript nor presented clearly as such in the results.  The second subquestion seems quite broad, and I’m not sure that the question actually anchors the inquiry; in such a case, one might expect to see studies organized by policymaking framework and discussed as such. Or else it might cover everything in this manuscript, in which case it is not a particularly useful research question to introduce alongside the others as it might subsume them. My point in this second critique is that the subquestions do not appear entirely to guide the work, and as such may set up the reader to expect something other than presented; I actually think that they could be excluded from the manuscript in favor of leaving a (revised) overarching question with the five motivating issues as a guide for the reader.

**Are sufficient details of the methods and analysis provided to allow replication by others?**

 I found the authors treatment of their “inductive qualitative approach” to be too thinly described.  It does not explain the extent to which their coding and subsequent analysis was guided by the motivating issues (5 of them, mentioned earlier in this review, which also appear to be an organizing feature of their results), nor what they actually did with the selection data about policy theory (see prior point about the first subquestion). I think this section could be better elaborated for replication but also for clarity and alignment among purposes, methods, results, and discussion.

**Is the statistical analysis and its interpretation appropriate?**

 No statistical analyses were conducted, nor would they be appropriate given the aims of this review.

 Under the Wider International Implementation Experiences section, there are many single paragraph cases illustrating a single study but each with its own header (beginning with The Lack of An Implementation Strategy). Given that the purpose of the review was to examine themes within the literature, I wonder if all of these separate headers are intended to constitute separate themes, and if so, whether they might be consolidated or otherwise more thoroughly discussed in terms of their distinctions. As is, it is hard to make connections among these various individual studies, and this section appears markedly different from prior sections which rarely summarize individual studies and more often synthesize them within headers that establish themes. If the authors are suggesting there are few common linkages and therefore each case makes its own unique contribution to the literature, that could be explained as well.

 Overall, the interpretation and synthesis of the literature was extremely well-written, informative, and useful.

**Are the conclusions drawn adequately supported by the results presented in the review?**

 I very much appreciate the following conclusion at the end of the international context section: *Consequently, equity policies focusing on social determinants, social justice, and inclusion, struggle to compete. They are overshadowed by more politically salient debates on the relationship between economic growth/ competitiveness and education, including the idea that we can quantify the relative performance of each country’s education system and use the data to improve each system ( Grek, 2009: 27; Rizvi & Lingard, 2010: 133–6). Almost all of these policies shelter under the umbrella term ‘education equity’ even if they achieve no such thing.* Actually, I very much appreciate many of the comments that point out the inherent tensions and unambiguous failure of neoliberal policies and assimilating/colonizing practices to address inequities globally.

 Aside from my appreciation of the authors’ articulation of the challenges and failures of educational equity policies, I found their discussion and conclusions to be very clearly based in the information presented in the Results section. 

 I did note that some equity research, however, does not appear in the results section and wondered if they might be integrated earlier on if they are to serve as examples of policy theory (e.g. Debray; Kretchmar). 

**Additional notes:**

- Under international agendas, the authors present OECD recommendations 1-5.  Under 3 are presumably 3 examples of such evidence-informed reform practices, but they are formatted in a way that makes it difficult to identify them as such.  The authors could better identify them as examples of recommendation 3. (They are excellent and useful examples by the way)
- Reference to Cairney’s blog (Methods) does not go to the correct link (though it does go to a very interesting article).
- HE is used without elaboration on the acronym

Are sufficient details of the methods and analysis provided to allow replication by others?

Partly

Are the rationale for, and objectives of, the Systematic Review clearly stated?

Partly

Is the statistical analysis and its interpretation appropriate?

Yes

Are the conclusions drawn adequately supported by the results presented in the review?

Yes

Reviewer Expertise:

Educational policy, evidence use, school improvement, school leadership

I confirm that I have read this submission and believe that I have an appropriate level of expertise to confirm that it is of an acceptable scientific standard, however I have significant reservations, as outlined above.

# Response

**Published:** 2021-12-22

**Authors:** Paul Cairney

**Affiliations:** University of Stirling, UK

Paul Cairney and Sean Kippin 22.12.21  p.a.cairney@stir.ac.uk Notes on the revision of ‘The future of education equity policy in a COVID-19 world: a qualitative systematic review of lessons from education policymaking’ https://open-research-europe.ec.europa.eu/articles/1-78/v1  

 We thank both reviewers for their thorough and constructive reviews, which helped us to improve our review. We summarise how the article has changed in response, then provide a full description of responses to each reviewer. Summary of changes:

1. Introduction: we (a) clarified the role of mainstream policy theory and (b) summarised the health equity strategy (HiAP) used as a comparison with education equity policy (pp3-6).
2. Methods: we clarified the (a) role of the IMAJINE project’s research questions and approach, (b) importance of immersion and induction, (c) coding, and (b) approach to analysis and synthesis (pp6-9).
3. Results: we altered subheadings and formatting to address the lack of clarity of some parts of the presentation. We edited this section to reduce the word count (to accommodate changes prompted by each reviewer) (pp10-35).
4. Conclusion: we reorganized and improved the discussion on policy and research implications (pp39-40).

**Dr Farley-Ripple**

1. *Clarify the central question and sub-questions*.
  We now make it clearer that, while we provide an overarching question – and set of sub-questions - to guide the IMAJINE project as a whole [e.g. Q: How does education equity research use policy theory to understand policymaking?], we adapt it to each individual review and try to explore the main question in that context. The latter requires some immersion in the field to avoid a too-rigid application of concepts from another discipline. To that end, we (a) adopt a low bar for inclusion (each article refers to at least one citation that discusses a policy concept) to (b) immerse ourselves in the field, and (c) provide a synthesis of the educational equity policy literature, which allows us to (d) provide an informed account of the – often limited - role of policy theory in analysis and explanation, and (e) reflect on the difference that engagement with policy theories makes. In other words, we wanted to show respect for the original aims of each article rather than fit them into too-rigid boxes in relation to policy theories.  One outcome of the review is the finding that, compared to our review of health equity (HiAP) research, education researchers draw less on ‘mainstream’ policy theories in favour of critical policy analysis and a sector-specific body of theory. This relative presence or absence is reflected in the word-count devoted to policy theories in each review.
2. *Provide a thicker, more convincing, description of ‘inductive qualitative approach’*.
  We clarify that our approach follows Sandelowski and Barroso’s (2007: xv) advice to foster respect for each author’s methods and aims while seeking to answer our guiding questions. We describe our approach in a new paragraph in Method [beginning **‘Fifth, we used an inductive qualitative approach to analyse each text, generate themes (Results), and relate them to policy theory insights (Discussion)’**].
3. *Clarify the use of so many sub-headings (in Wider International Implementation Experiences) and consider dropping them*.
  We replace a series of headings with two sections introduced via an in-text summary.
4. *Consider putting the articles that cite policy theories in the main results section* (Debray; Kretchmar).
  This issue arises partly from our use of standard headings of systematic reviews (which separates Results from Discussion in a way that is not entirely helpful to us) and the value of emulating the structure of the previous (HiAP) review. The Discussion section consists of summarized and synthesized insights from the articles that draw relatively sparingly on policy theories. The Results section focuses on the (relatively few) articles that engage directly with mainstream policy theories, which we use to produce key insights from a combined focus on policy theories and specialist education research. We make this distinction clearer in the Introduction, particularly in the revised final paragraph **: ‘Our Discussion section relates these Results to the three key insights – on policy change, bounded rationality, and policymaking complexity – that derive from policymaking concepts and theories. We describe these general insights more fully and show how a small subset of included articles uses them to explain education policy dynamics. These articles show how policy concepts and theories inform the study of education equity policy. First, they highlight the general difference between education equity policy on paper and in practice. Second, they show how policymakers deal with bounded rationality by: (a) paying minimal attention to key equity issues; (b) relying on actors who share their beliefs; (c) emulating other governments without understanding their alleged success; and (d) basing policy on social stereotypes, while (e) describing their choices as ‘evidence based’. Third, they explain how complex policymaking environments mediate policy change. Overall, these insights contribute to a commonly told story in education equity research: there is high rhetorical but low substantive commitment to reducing unfair inequalities, and the dominant neoliberal approach undermines the social justice approaches that are essential to policy progress’.**

We also addressed the formatting, headings, and weblink issues described by Dr Farley-Ripple.
